# Genome-centered metagenomics illuminates adaptations of core members to a partial Nitritation–Anammox bioreactor under periodic microaeration

**DOI:** 10.3389/fmicb.2023.1046769

**Published:** 2023-01-26

**Authors:** Yung-Hsien Shao, Yu-Wei Wu, Muhammad Naufal, Jer-Horng Wu

**Affiliations:** ^1^Department of Environmental Engineering, National Cheng Kung University, Tainan, Taiwan; ^2^College of Medical Science and Technology, Graduate Institute of Biomedical Informatics, Taipei Medical University, Taipei, Taiwan

**Keywords:** nitrogen removal, single-stage partial nitritation-anammox, metagenomics, comammox, anammox

## Abstract

The partial nitritation-anaerobic ammonium oxidation (anammox; PN-A) process has been considered a sustainable method for wastewater ammonium removal, with recent attempts to treat low-strength wastewater. However, how microbes adapt to the alternate microaerobic-anoxic operation of the process when treating low ammonium concentrations remains poorly understood. In this study, we applied a metagenomic approach to determine the genomic contents of core members in a PN-A reactor treating inorganic ammonium wastewater at loading as low as 0.0192 kg-N/m^3^/day. The metabolic traits of metagenome-assembled genomes from 18 core species were analyzed. Taxonomically diverse ammonia oxidizers, including two *Nitrosomonas* species, a comammox *Nitrospira* species, a novel *Chloroflexota*-related species, and two anammox bacteria, *Ca.* Brocadia and *Ca.* Jettenia, accounted for the PN-A reactions. The characteristics of a series of genes encoding class II ribonucleotide reductase, high-affinity *bd*-type terminal oxidase, and diverse antioxidant enzymes revealed that comammox *Nitrospira* has a superior adaptation ability over the competitors, which may confer the privileged partnership with anammox bacteria in the PN-A reactor. This finding is supported by the long-term monitoring experiment, showing the predominance of the comammox *Nitrospira* in the ammonia-oxidizing community. Metagenomic analysis of seven heterotrophs suggested that nitrate reduction is a common capability in potentially using endogenous carbohydrates and peptides to enhance nitrogen removals. The prevalence of class II ribonucleotide reductase and antioxidant enzymes genes may grant the adaptation to cyclically microaerobic/anoxic environments. The predominant heterotroph is affiliated with Chloroflexota; its genome encodes complete pathways for synthesizing vitamin B6 and methionine. By contrast, other than the two growth factors, *Nitrospira* and anammox bacteria are complementary to produce various vitamins and amino acids. Besides, the novel *Chloroflexota*-related ammonia oxidizer lacks corresponding genes for detoxifying the reactive oxygen species and thus requires the aid of co-existing members to alleviate oxidative stress. The analysis results forecast the exchanges of substrates and nutrients as well as the collective alleviation of oxidative stress among the core populations. The new findings of the genomic features and predicted microbial interplay shed light on microbial adaptation to intermittent microaeration specific to the PN-A reactor, which may aid in improving its application to low-strength ammonium wastewater.

## 1. Introduction

Driven by the demand for energy-neutral wastewater treatment, the partial nitritation-anaerobic ammonium oxidation (anammox; PN-A) process has received considerable attention (Lackner et al., 2014). This biotechnology has been described as a sustainable method of wastewater ammonium removal because of its relatively low energy consumption, low production of wasted sludge, and lack of a need for organic carbon supplementation. Nitrogen removal through PN-A proceeds in two steps: (1) ammonia-oxidizing microorganisms oxidize approximately 57% of input ammonium to nitrite, and (2) anammox bacteria convert the unreacted ammonium and nitrite into dinitrogen (N_2_). During the process, nitrate accumulates as a byproduct from the anammox or nitrite-oxidizing bacteria (NOB). Although the PN-A process has been successfully implemented to treat high-strength ammonium wastewater (>500 mg-N/L) worldwide (Lackner et al., 2014), its applications to low-strength wastewater, such as municipal wastewater (20 to 60 mg NH_4_^+^-N/L) (Wang et al., 2022), are still challenging (Cao et al., 2017; Qiu et al., 2021). Because mainstream wastewater accounted for the majority of N load in a wastewater treatment plant, extensive work has been recently dedicated to the PN-A treatment (Wang et al., 2022).

Oxygen supply is one of the most critical challenges in managing PN-A systems treating low-strength ammonium wastewater (Yu et al., 2022). PN-A systems are usually operated in a single reactor (Lackner et al., 2014), in which effective nitrogen removal relies on the close cooperation of aerobic and anaerobic microbes. A proposed strategy for oxygen control is intermittent microaeration, which implements short aeration duration with a low amount of oxygen (microaeration) but avoids inhibiting the anammox activity and producing nitrate from NOB. Intermittent microaeration separates the redox conditions into cyclically microaerobic (aerated) and anoxic (nonaerated) periods. Limiting the oxygen supply reduces the nitrification efficiency (ammonia and nitrite oxidations). This usually leads to inadequate ammonia-oxidizing activities in the mainstream PN-A systems with ammonium accumulation in the effluent (Lotti et al., 2015; Kouba et al., 2016; Wu et al., 2021). Anammox activity is sensitive to oxygen and can be completely inhibited at dissolved oxygen (DO) <0.04–0.12 mg/l (Strous et al., 1997; Egli et al., 2001; Oshiki et al., 2016). It has been reported that anammox bacteria were lost over time with DO concentrations of 0.2–1.0 mg/l, which deteriorated the nitrogen removal performance of a mainstream PN-A bioreactor (Roots et al., 2019). Although studies have reported that the preferred DO levels for mainstream PN-A were lower than 0.5 mg/l (Li et al., 2016; Akaboci et al., 2018; Xu et al., 2020), the effective nitrogen removal performance could be achieved with a wide DO range from lower than 0.5 to higher than 1.0 mg/l (see reviews by Miao et al., 2022). In addition to the differences in biomass types (suspended sludge, biofilm, and granule) and reactor configurations, understanding the underlying rationale of how PN-A populations adapt to the periodical microaeration is essential for optimizing PN-A systems.

In addition to autotrophic nitrifying and anammox populations, 16S rRNA gene-based analyzes have indicated the presence of heterotrophic bacteria of phyla *Chloroflexota*, *Planctomycetota*, *Bacteroidota*, *Proteobacteria*, *Acidobacteriota,* and *Armatimonadota* with a considerable proportion across different anammox-associated systems (Pereira et al., 2017; Zhu et al., 2017). Various cross-feeding ecological scenarios involving anammox and heterotrophic bacteria have been proposed based on the functional prediction of metagenomic analysis (Speth et al., 2016; Lawson et al., 2017; Zhao et al., 2018; Wang et al., 2019; Ji et al., 2021). For example, heterotrophic bacteria could scavenge soluble microbial products and extracellular polymeric substances (EPS) produced by anammox bacteria for their growth (Lawson et al., 2017; Wang et al., 2019). Anammox bacteria are likely to supply specific amino acids and vitamins to the auxotrophic heterotrophs, which lack biosynthesis pathways for specific nutrient compounds (Lawson et al., 2017; Ji et al., 2021). Conversely, *Armatimonadota* and *Proteobacteria* bacteria are likely to provide secondary metabolites such as folate and molybdopterin to anammox bacteria, which lack these biosynthesis pathways (Zhao et al., 2018). In addition, heterotrophic bacteria could benefit anammox bacteria through the production of nitrite from partial denitrification and dissimilatory nitrate reduction to ammonium (DNRA; namely, the nitrite loop) (Speth et al., 2016; Lawson et al., 2017; Wang et al., 2019; Ji et al., 2021). However, previous studies have mainly focused on PN-A systems treating high-strength ammonium wastewater (Speth et al., 2016; Wang et al., 2019). The microbial composition and functional traits in PN-A systems receiving low-strength wastewater in the context of microaeration remain insufficient.

Previous metagenomic studies of anammox-associated systems have mainly emphasized nitrogen- and carbon-related cross-feeding between anammox and heterotrophic bacteria (Speth et al., 2016; Wang et al., 2019) but have overlooked how the microbes adapt to the PN-A-specific intermittent microaeration. This study operated an intermittently aerated bioreactor performing the PN-A process with low ammonium loading (19.2 mg-N/L/day). The metagenomic approach was utilized to decipher the genomic content of core members that exhibited ecological success in this system. We investigated the distribution of genes related to nitrogen, carbon, and oxygen metabolisms in the draft genomes of the core members. Subsequently, the dynamics of the potential ammonia-oxidizing members were investigated. Our results shed light on the adaptation of core microbes to the distinctive redox environments in the PN-A reactor and may contribute to applications of PN-A systems for treating low-strength ammonium wastewater.

## 2. Materials and methods

### 2.1. Sludge samples collection, DNA extraction, and sequencing

A lab-scale PN-A bioreactor in the chemostatic mode was operated under low DO conditions to treat synthetic inorganic ammonium wastewater (Shao and Wu, 2021). Sludge samples were taken from the PN-A bioreactor on days 191 and 235 for shotgun metagenomic and 16S rDNA amplicon sequencing. The samples were filtered through a 0.22-μm membrane to harvest microbial cells. Subsequently, total DNA was extracted using the DNeasy PowerWater kit (Qiagen, Hilden, Germany) according to the manufacturer’s instructions. For shotgun metagenomic sequencing, DNA library preparation and sequencing on the Illumina HiSeq platform were performed by Welgene Biotech (Taipei, Taiwan) to generate 2 × 150 bp paired-end reads. For 16S rDNA amplicon sequencing, library preparation of the hypervariable V3-V4 fragment regions and 2 × 300 bp paired-end sequencing on the Illumina MiSeq platform were performed by Genomics, BioSci & Tech (New Taipei City, Taiwan). The details of 16S rDNA amplicon sequencing data processing are reported in the Supplementary methods (Shao and Wu, 2021).

### 2.2. Metagenomic analysis

The retrieved reads from the two DNA samples were trimmed using Trimmomatics v0.39 (parameter: ILLUMINACLIP:TruSeq3-PE.fa LEADING:12 TRAILING:12 SIDINGWINDOW:4:15 MINLEN:36) (Bolger et al., 2014) and co-assembled using metaSPAdes v3.13.0-dev (Nurk et al., 2017) with parameter–meta, followed by automated binning using MaxBin 2.0 with default settings (Wu et al., 2016) to generate metagenome-assembled genomes (MAGs). The quality of recovered MAGs was estimated using CheckM version 1.1.3 (Parks et al., 2015). The relative abundance of MAGs was calculated according to the method reported previously using bbmap version 38.90^1^ with the parameters *minid* = 0.95, *ambig1* = random, and *ambig2* = best (Zhao et al., 2018). Briefly, the clean reads from each sample were mapped to all contigs assembled to the draft genomes; then the reads per kilobase per million mapped reads (RPKM) of each MAG was calculated as the numbers of clean reads from a sample mapped to a MAG divided by its genome size (per kilobase) and the total number of mapped reads of that sample (per million). The relative abundance was calculated as the RPKM value of each MAG divided by the sum of the RPKM values of all draft genomes in each sample, and then multiplied the percentage of mapped reads of the sample.

For a phylogenomic analysis of MAGs, GTDB-Tk v1.5.0 with reference database (GTDB R06-RS202) was used to determine the genomic taxonomy (Chaumeil et al., 2019), and Average Nucleotide Identity (ANI) between query and their closest reference genome identified by GTDB-Tk was calculated by using FastANI (Jain et al., 2018) as a dependency of GTDB-Tk. Subsequently, MAGs and their closest reference genome were subjected to a phylogenetic tree construction based on a set of HMM profiles for 74 bacterial single-copy genes using GToTree version v1.5.1 (Lee, 2019) and the dependencies HMMER3 v3.3.2 (Eddy, 2011), MUSCLE v3.8.1551 (Edgar, 2004), TrimAl v1.4.rev15 (Capella-Gutierrez et al., 2009), Prodigal v2.6.3 (Hyatt et al., 2010), and FastTree 2 v2.1.10 (Price et al., 2010). The phylogenetic tree was then visualized using iTOL (Letunic and Bork, 2021).

For functional annotation, open reading frames (ORFs) of each MAG were first predicted using Prokka v1.13.4 (Seemann, 2014) and annotated using GhostKOALA (Kanehisa et al., 2016) for the assignment of KO identifiers (K number) and KEGG Mapper (Kanehisa and Sato, 2020) for the reconstruction of specific metabolic pathways in the KEGG module. Additionally, ORFs were analyzed using BLASTP searches against the NCBI nr database with an E value of <10^−5^. The inconsistent annotation results of ORFs between BLASTP and GhostKOALA predictions were then confirmed using a phylogenetic tree analysis with reference sequences. To explore the potential activity of exoenzymes, genes encoding the carbohydrate-active enzyme peptidase and transporter were identified using BLASTP searches with an E value of <10^−100^ against the Carbohydrate-Active enZYmes Database (CAZyDB.07312020) (Drula et al., 2022), MEROPS release 12.3 (Rawlings et al., 2018), and the Transporter Classification Database (Saier et al., 2021). The subcellular location of identified CAZymes and peptidases was predicted using CELLO v.2.5 (Yu et al., 2004). A hierarchical clustering was performed to compare the potential of the core MAGs for adaptation to periodic microaeration using the pheatmap package in the R platform (version 3.6.3) (R Development Core Team, 2020).

To determine the microbial composition, a taxonomic classification of retrieved reads was performed using Kaiju v1.8.2 (in the heuristic Greedy mode allowing up to 5 mismatches) (Menzel et al., 2016), with the NCBI nr database (rel. 84) as the reference to determine the microbial community composition.

### 2.3. Phylogenetic analysis of functional genes

The ORFs predicted to encode particulate methane monooxygenase (pmoA)/ammonia monooxygenase (amoA), ribonucleotide reductase, and terminal oxidase by GhostKOALA were selected for phylogenetic analysis. These ORFs and reference sequences were aligned using MUSCLE (Edgar, 2004) at the default settings. Then, maximum likelihood phylogenetic trees were constructed using MEGA7 (Kumar et al., 2016) with 1,000 replications in the bootstrap test.

### 2.4. Primer design and quantitative polymerase chain reaction

To assess how genetic differences among potential ammonia oxidizers may influence their temporal dynamics, we designed oligonucleotide primers targeting the *amo* sequences from the MAGs (*amoA* of NTP03 and CFX14 and *amoC* of PRO01 and PRO05) of this study. The details of primer design and validation are presented in the Supplementary methods. Quantitative polymerase chain reaction (qPCR) was performed to determine the concentrations of *amo* genes of potential ammonia oxidizers according to previously reported protocols (Shao and Wu, 2021).

## 3. Results

### 3.1. Reactor operation and microbial community

The bioreactor fed with synthetic inorganic wastewater containing ammonium (120–220 mg-N/L) as the sole substrate was operated for cultivating anammox and nitrifying populations under low DO conditions. The intermittent microaeration was applied to achieve a DO cycling from about 0.5 mg/l during microaeration to <0.1 mg/l without an oxygen supply. For metagenomic analysis, two sludge samples were taken on days 191 and 235 when the reactor operation was maintained at a stable nitrogen removal efficiency of 67.7 ± 5.2% in the period. During this period, ammonium and nitrite were not detected in the effluent, but nitrate accumulated with average concentrations of 37.7 ± 6.6 mg-N/L, in which about 30 and 70% resulted from anammox and nitrite oxidation, respectively. Anaerobic batch tests validated that denitrification was negligible in the reactor (Shao and Wu, 2021), suggesting nitrogen loss was predominantly contributed by PN-A reactions.

Microbial community compositions were determined using 16S rDNA amplicon sequencing and shotgun metagenomic analyzes. Combined results from the two methods indicated the dominance of phyla *Proteobacteria* and *Planctomycetota* in both samples (Supplementary Figure S1). Species of the phyla *Bacteroidota, Nitrospirota,* and *Chloroflexota* were abundant, but varied abundances were detected using different methods. *Bacteroidota* indicated an abundance of 12.5–12.8% in the 16S rRNA gene amplicon sequencing results, but was relatively rare (1.7–2.5%) in the shotgun metagenomic analysis results. These discrepancies were likely due to both uneven amplification of 16S rRNA and differences in the analytical methods. In addition to minor anammox genera, such as *Candidatus* Kuenenia and *Candidatus* Brocadia, the anammox genus *Candidatus* Jettenia (amplicon, 20.5–32.2%; metagenomics, 8.4–11.4%) and nitrifying genera *Nitrospira* (amplicon, 7.1–7.4%; metagenomics, 7.7–8.0%) and *Nitrosomonas* (amplicon, 15.0–16.9%; metagenomics, 1.8–2.4%) were highly dominant in the PN-A reactor (Supplementary Table S1), accounting for the observed nitrification and anammox performance. Notably, a large proportion of clean reads could not be assigned to any known phylum (34–41%) or any genus (67–71%) in the metagenomic sequencing dataset, indicating the low resolution of read-based metagenomic analysis in determining the overall microbial composition of the PN-A reactor of this study.

### 3.2. Relative abundance and phylogeny of the core MAGs

Clean reads with an average quality score > 30 from the two samples were co-assembled and binned, resulting in 162 MAGs. After CheckM analysis, 85 high- or medium-quality draft genomes (completeness >75% and contamination <10%; see Supplementary Data 1 for the genome statistics) were then selected for analysis of relative abundance and function profiling. A total of 18 MAGs were defined as core MAGs if they qualify any of the two criteria: (1) occurred in both samples with abundance >1% in any sample (Galand et al., 2009); or (2) consisting of genes related to PN-A reactions (*amo* and *hdh*). The genome statistics are summarized in Supplementary Table S2.

**Figure 1:**
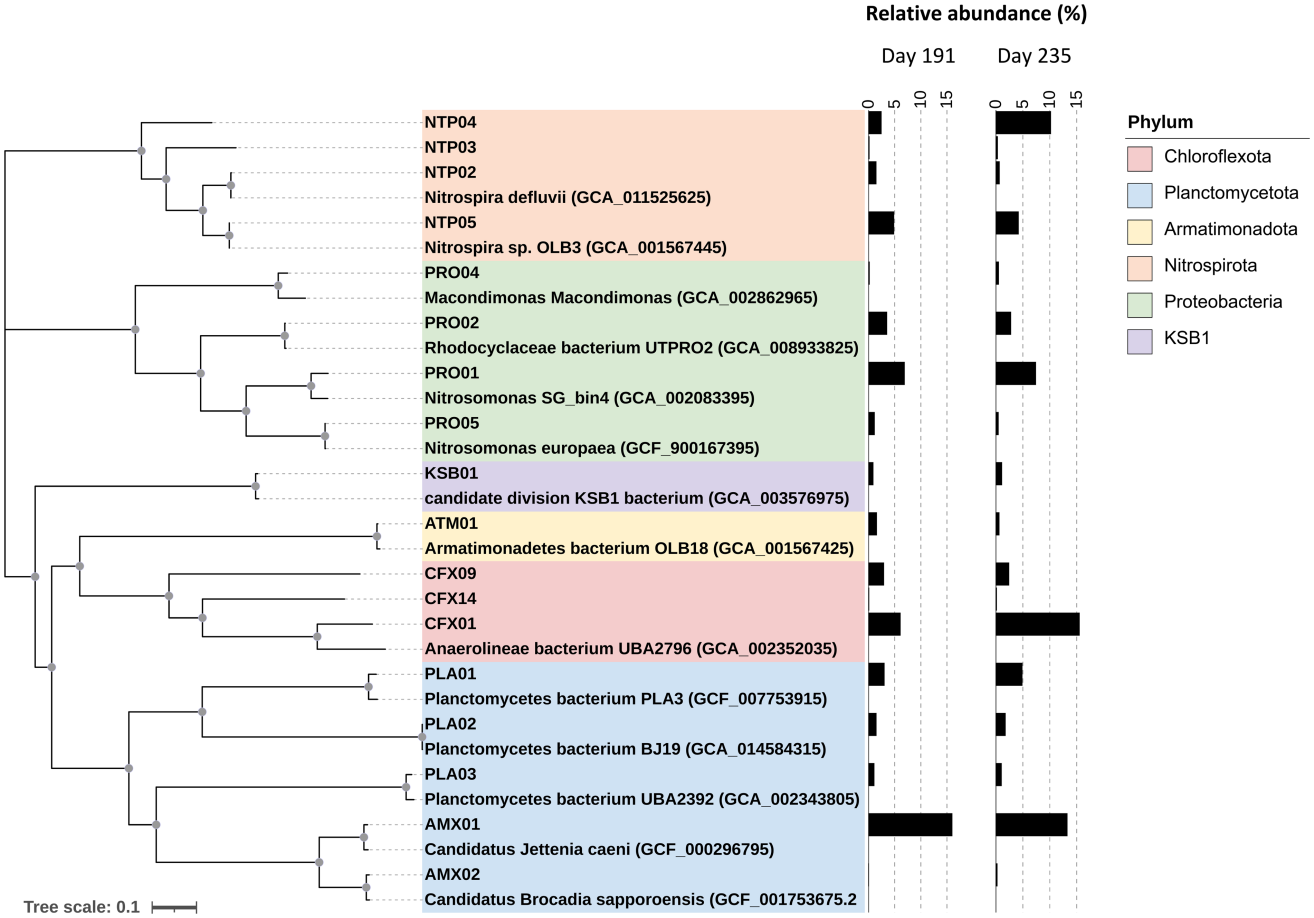
Phylogenomic analysis of 18 core MAGs based on the amino acid sequence of 34 single-copy marker genes. The phyla-taxa of these MAGs are indicated by colored boxes and their relative abundances are shown in the bar plot. The nodes with a bootstrap value higher than 90% are indicated as grey solid dots.

Figure 1 illustrates the phylogenomic tree of core MAGs with their relative abundance. These 18 core MAGs accounted for a total abundance of 49.4 and 61.5% with respect to the total number of clean reads on days 191 and 235, respectively, covering predominant bacterial species in the reactor. AMX01 with an ANI of 94.5% was related to *Candidatus* Jettenia and dominated the community at an abundance of 12 to 16%, and another anammox MAG, AMX02, with a 96.5% ANI similarity to *Candidatus* Brocadia only accounted for less than 0.2% in both samples. Interestingly, the abundance (15%) of CFX01 exceeded AMX01 on day 235 as the most abundant species. CFX01 was heterotrophic and affiliated with phylum *Chloroflexota* but only shared an average nucleotide identity of 76.8% to its closest reference genome, *Anaerolineales* bacterium UBA2796 (Supplementary Table S2). The third dominant MAG was affiliated with the genus *Nitrosomonas* (PRO01), which was regarded as AOB. We obtained four core MAGs belonging to the genus *Nitrospira,* which accounted for 8.3 and 14.4% of the whole community on days 191 and 235, respectively. Additional heterotrophic MAGs with lower abundance were detected, including two *Chloroflexota* MAGs (CFX09 and CFX14), three *Proteobacteria* MAGs (PRO02, PRO04, and PRO05), three *Planctomycetota* MAGs (PLA01, PAL02, and PLA03), and one *Armatimonadota* (formally candidate phylum OP10) MAG (ATM01). A high-quality MAG KSB01 with a genome size of approximately 2.2 Mb and an average abundance of 0.92% was assigned to the candidate phylum KSB1.

### 3.3. Nitrogen metabolism

**Figure 2:**
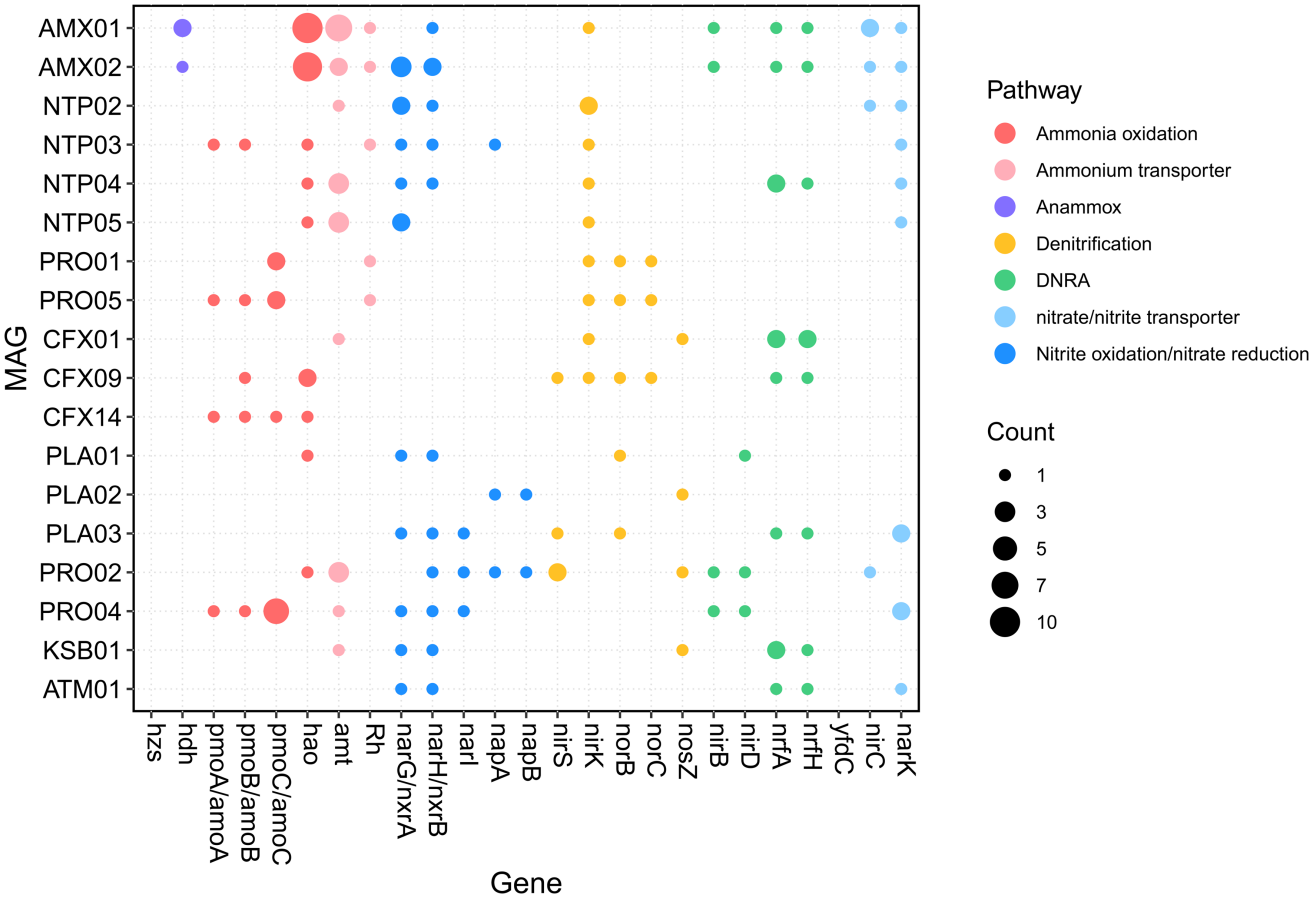
Distribution of the nitrogen-converting functional genes retrieved from the 18 core MAGs.

We first analyzed the potential for nitrogen conversion of core MAGs (Figure 2). The details of analyzed KO are described in Supplementary Table S3 and the overall KEGG annotation with corresponding blastp search results of interested KO are presented in Supplementary Data 2. In total, four MAGs, namely PRO04, PRO05, NTP03, and CFX14, harbored ORFs predicted as *amoA*/*pmoA* based on the ko number assigned in GhostKOALA; this suggests that a variety of MAGs were responsible for ammonia oxidation in our reactor. A phylogenetic analysis of these ORFs was performed to distinguish *amoA* from *pmoA* (Supplementary Figure S2). The *amoA* of PRO05 was clustered within *amoA* of *Nitrosomonas*; this is consistent with the taxonomic classification on the basis of 34 bacterial single-copy genes (Figure 1). The NTP03 *amoA* was clustered with the comammox *amoA* clade A, suggesting that NTP03 is a complete ammonia-oxidizing *Nitrospira* (Daims et al., 2015; van Kessel et al., 2015). Compared with reference to the high-quality comammox genomes from public databases (Supplementary Table S4), NTP03 encoded a variety of genes related to the decomposition of organic nitrogen, including *ure*, *fdhA*, *cynS,* and *amiE* (Supplementary Figure S3). Notably, the *amiE* gene, which encodes the amidase catalyzing the hydrolysis of amide-related compounds, was only observed in NTP03 among all the analyzed comammox genomes. Unexpectedly, *amoABC* and *hao* were observed in *Chloroflexota*-affiliated CFX14. The *amoA*, *amoB*, and *amoC* shared a sequence similarity of 94.9, 95.7, and 94.5%, respectively, with the counterparts of *Nitrosospira multiformis* ATCC 25196 (CP000103.1). However, 16S rDNA amplicon sequencing indicated an absence of the genus *Nitrosospira* in both samples (Supplementary Table S5). A PCR experiment with the primer sets [NSS-209F and NSS-478R (Lim et al., 2008)] specific to the 16S rRNA gene of *Nitrosospira* failed to generate the amplicon products with the DNA of the two samples, confirming the absence of *Nitrosospira*. These findings suggest that in addition to the three known ammonia oxidizers, two *Nitrosomonas* genomes (PRO01 and 05), and one comammox *Nitrospira* (NTP03), a new ammonia oxidizer, CFX14, potentially contributed to ammonia oxidation in the reactor. Two MAGs, AMX01 and AMX02, encoded *hdh* for oxidation of hydrazine to N_2_ but lacked *hzs* according to the annotation of the KEGG classification system. The blastp search against the nr database detected an ORF of AMX01 (DMEBDDEC_02210) sharing a 97.9% identity with *hzsC* from *Candidatus* Jettenia ecosi (TLD39959.1). For the nitrite oxidation, four *Nitrospira*-related genomes were recovered. Three of them (NTP02, NTP04, and NTP05) only harbored *nxr* and thus are strict nitrite-oxidizing *Nitrospira*.

Genes encoding the capability to gain energy by reducing nitrogen oxides were commonly distributed in the heterotrophic bacteria in this PN-A reactor. Genes involved in denitrification were present in all heterotrophic MAGs except CFX14. Notably, among four potential ammonia oxidizers, only *Nitrosomonas* MAGs (PRO01 and PRO05) encoded *norBC* for the production of nitrous oxide. Genes encoding nitrite reductase for reducing nitrite to ammonia through DNRA were also prevalent in heterotrophic MAGs. In addition, anammox AMX01 and AMX02 and *Nitrospira* NTP04 also harbored genes, such as *nirB*, *nrfA,* and *nrfH,* involved in DNRA. These functions may jointly facilitate the removal of nitrate produced by NOB and anammox bacteria.

### 3.4. Carbon metabolism

**Figure 3:**
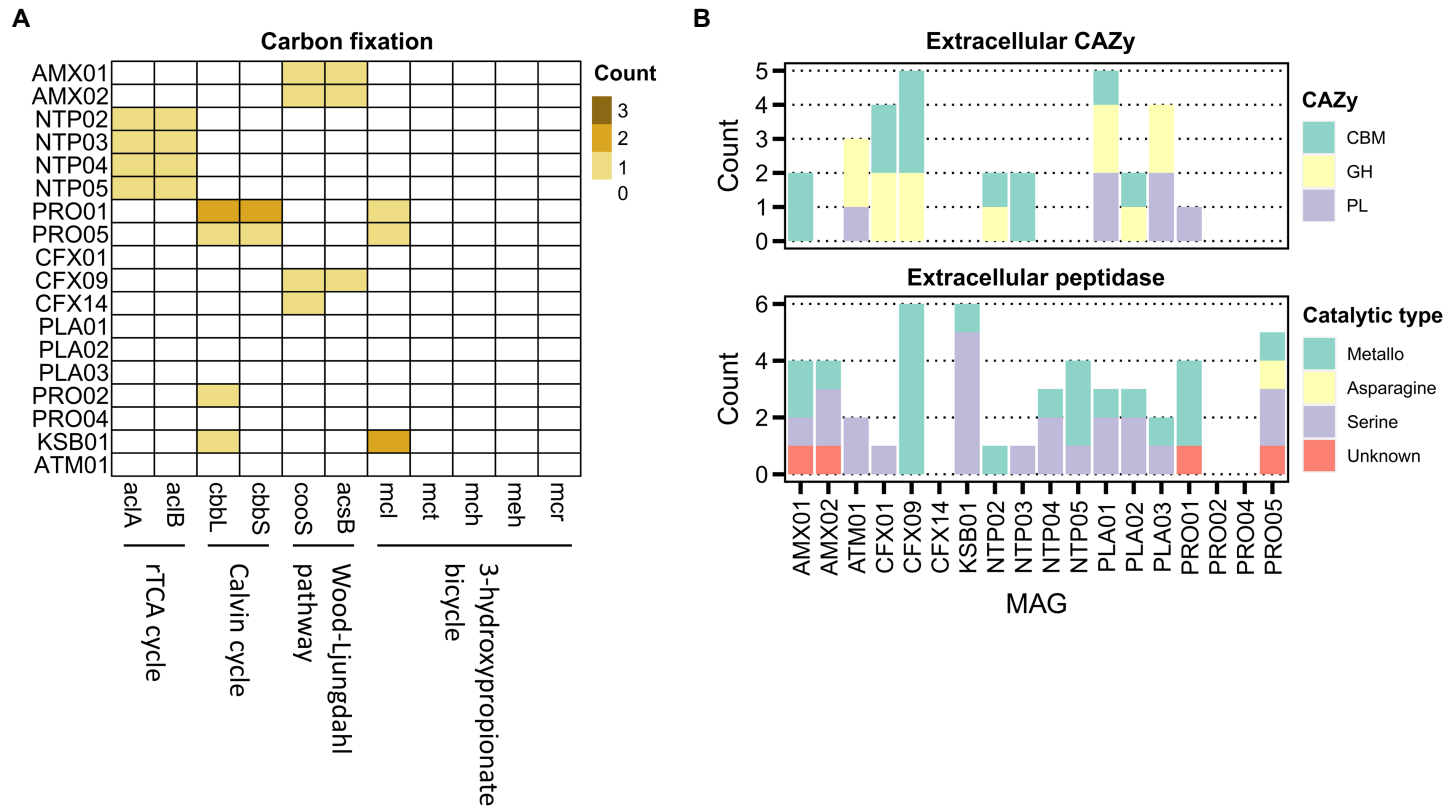
Distribution of functional genes for the carbon fixation **(A)**, and the genes encoding extracellular carbohydrate-active enzymes (CBM, carbohydrate-binding module; GH, glycoside hydrolases; PL, polysaccharide lyases) and peptidases **(B)** in the core MAGs.

Central carbon metabolic pathways were common in most core MAGs with a high level of completeness (complete or only one block missing; Supplementary Figure S4A). Because carbon metabolism was driven by the fixation of carbon dioxide in the reactor, we then examined the four detected carbon fixation pathways using key target genes (Figure 3A; Supplementary Table S3). The results suggested that anammox, *Nitrospira,* and *Nitrosomonas* MAGs could fix inorganic carbon through the Wood–Ljungdahl pathway, *r*TCA cycle, and Calvin cycle, respectively. Moreover, CFX09 could be autotrophic since this MAG harbored marker genes and a nearly complete module of the Wood–Ljungdahl pathway with only one missing block, the *fhs* gene-encoding formate-tetrahydrofolate ligase.

We then investigated the potential of the microbiome for the degradation of extracellular carbohydrates and peptides, which were the proposed substrate for the growth of heterotrophs (Figure 3B). Only several denitrifying MAGs, mainly affiliated with *Chloroflexota* and *Planctomycetota*, encoded glycoside hydrolases and polysaccharide lyases, predicting the ability of the extracellular carbohydrate degradation. The extracellular peptidases of the serine- and metallo-superfamilies were abundant in most of the core MAGs, suggesting a common feature of extracellular protein decomposition. Further analysis of genes encoding transporters for carbohydrates, peptides, and amino acids indicated the prevalence of these genes in most heterotrophic MAGs (Supplementary Data 2), including the MAGs lacking the ability to produce extracellular CAZys and peptidases (PRO02, PRO04, and CFX14). These findings suggest cross-feeding not only between heterotrophs and autotrophs but also between heterotrophs.

Because heterotrophs might conserve energy from fermentation, we investigated the potential for formate, lactate, ethanol, and acetate fermentation. The functions for acetate production were annotated in PRO02, PRO05, and KSB1 (Supplementary Figure S4B). The produced acetate could be incorporated into biomass through the acetyl-CoA synthetase (acs) observed in 17 core MAGs (except for CFX14). Besides, ATM01 harbored *ldh*, which encodes *L*-lactate dehydrogenase for lactate fermentation (Supplementary Data 2).

It has been recognized that cross-feedings of amino acids and vitamins were essential for auxotrophs in the anammox-associated systems (Lawson et al., 2017). We thus studied the amino acid and vitamin biosynthesis with the module completeness in the pathways illustrated in Supplementary Figure S5. Anammox bacteria, *Nitrosomonas* and *Nitrospira* harbored pathways (complete and one block missing) for synthesizing most amino acids with the exception of methionine. By contrast, the potentials of amino acid biosynthesis varied among the heterotrophs. Only several members of *Chloroflexota* (CFX01 and CFX09) and *Proteobacteria* (PRO02 and PRO04) possessed the biosynthesis pathway for most amino acids. The others could be auxotrophic in view of the incompleteness pathway of specific amino acids. For vitamin biosynthesis, most heterotrophic MAGs lacked pathways for the biosynthesis of thiamine (vitamin B1) and cobalamin (vitamin B12), but the autotrophic members exhibited all these corresponding capabilities. Notably, most of the core members have poor abilities for pyridoxal phosphate (vitamin B6) synthesis, except for the three MAGs (CFX01, CFX09, and ATM01). As the high abundance in the reactor, the *Chloroflexota* CFX01 could play a vital role in supplying pyridoxal phosphate in the PN-A reactor. As a whole, the different potentials for the production of amino acids and vitamins were complementary among microbial community members; this indicates that species are dependent on one another to obtain essential nutrients.

### 3.5. Oxygen-related adaptations

**Figure 4:**
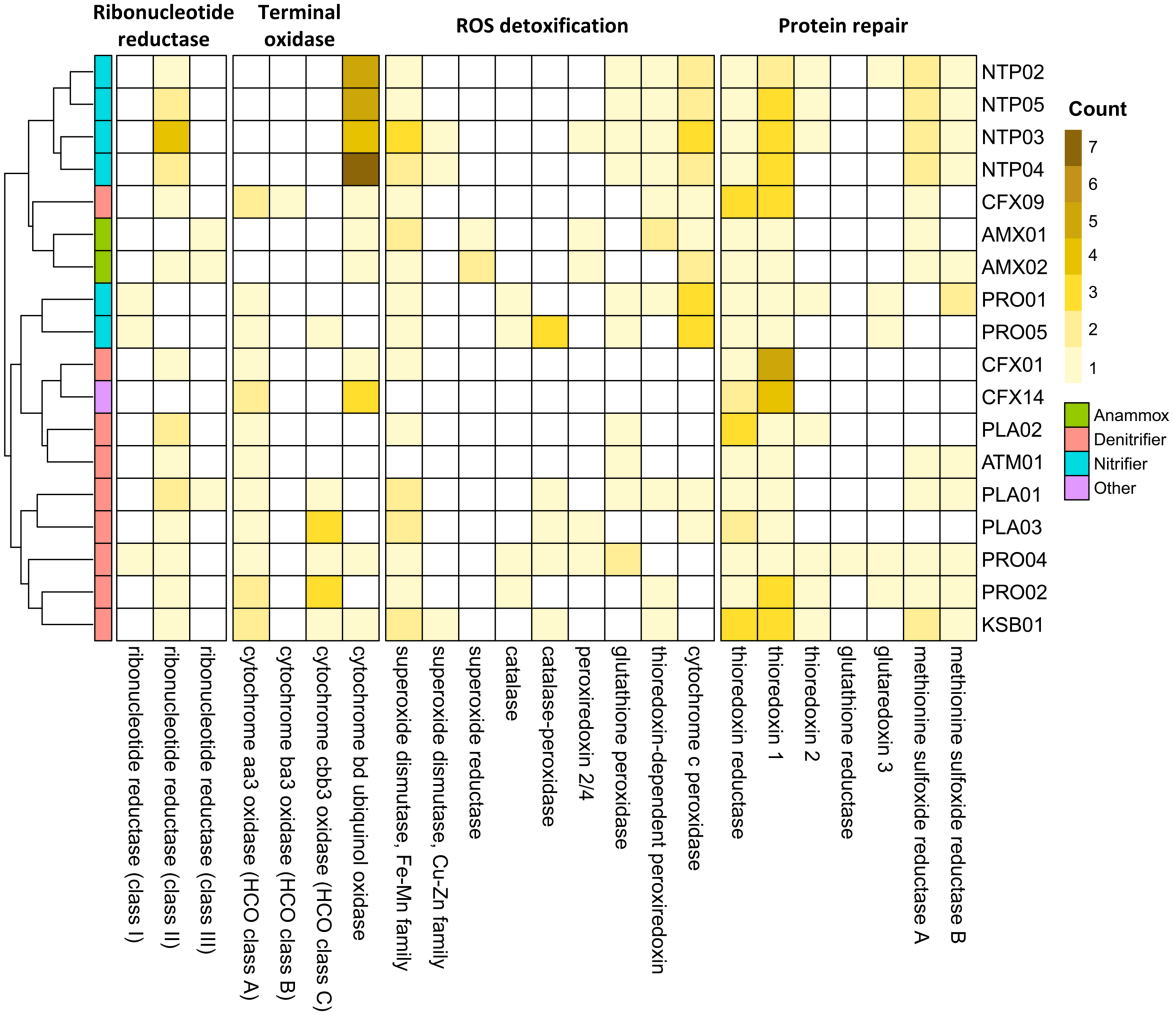
Distribution of genes encoding ribonucleotide reductases, terminal oxidases and ROS defense enzymes detected in the 18 core MAGs.

To study the bacterial abilities to adapt to microaerobic-anoxic cycling in the PN-A reactor, we explored a series of specific genes encoding ribonucleotide reductase (RNR) and terminal oxidase, ROS detoxification, and protein repair. The annotated subclassification of genes encoding ribonucleotide reductase and terminal oxidase was validated using phylogenetic analysis (Supplementary Figures S6, 7; Supplementary Table S6). Figure 4 shows the cluster analysis of the four gene groups. In general, nitrifying and anammox MAGs were clustered with their phylogenetic counterparts, while denitrifying MAGs were divided into two main clusters. The clustering analysis revealed a consistent tendency of *Nitrosomonas*, *Nitrospira*, and anammox MAGs with aerobic, microaerobic, and anoxic lifestyles, respectively. The heterotrophs PLA01, PLA03, PRO02, PRO04, and KSB01 formed a cluster because they shared a similar gene distribution with the oxygen-dependent class II RNR, high-affinity terminal oxidase, ROS-detoxifying, and protein-repairing enzymes, for their adaptations to the periodic microaeration of the PN-A reactor. Another cluster comprising CFX01, CFX14, PLA02, and ATM01 might be less adaptive due to the lack of most genes encoding the antioxidant enzyme.

The RNR that converts ribonucleotides to deoxyribonucleotides used by an organism reflects its potential for aerobic and anaerobic physiology (Poole et al., 2002). Three RNR classes have been identified. Class I RNRs require O_2_ for activation, thus aerobic. The RNRs in class III are sensitive to O_2_ and only active under anaerobic conditions. The class II RNR is oxygen-independent (facultative) and relies on adenosylcobalamin (vitamin B_12_) for proper functionality (Torrents, 2014). As shown in Figure 4, *Nitrosomonas* MAGs (PRO01 and PRO05) only encoded class I RNRs, while the class III-RNR genes were detected in two anammox MAGs. These observations agreed with the aerobic and anaerobic lifestyles of *Nitrosomonas* and anammox bacteria, respectively. All denitrifying and *Nitrospira* MAGs harbored class II-RNR genes, revealing their survival in the alternate transition from microaerobic to anoxic environments. Still, the *Ca.* Brocadia AMX02 and *Planctomycetota* PLA01 possessed multiple types of RNR genes, conferring a wide redox range for deoxyribonucleotide biosynthesis.

The distribution of genes encoding terminal oxidase, including low-affinity terminal oxidase (*aa_3_*-type) and high-affinity terminal oxidase (*ba_3_*, *cbb*_*3*,_ and *bd*-type), are shown in Figure 4. Most core members harbored more than one high-affinity terminal oxidase. Notably, *Nitrospira* MAGs have 4–7 copies of high-affinity *bd*-type terminal oxidases, which is higher than other core members (1 ~ 3 copies). This finding highlights the excellent ability to oxidize ammonia and nitrite in oxygen-limited conditions. By contrast, *Armatimonadota* ATM01, *Planctomycetota* PLA02, and *Nitrosomonas* PRO01 only exhibited low-affinity terminal oxidases (*aa_3_* type), suggesting these species might be less competitive in utilizing oxygen at low concentrations.

In accordance with the genes involved in ROS detoxification (Johnson and Hug, 2019) and the repair of oxidatively damaged proteins (Ezraty et al., 2017), a total of 16 relevant genes were detected with the core MAGs (Figure 4). Superoxide dismutase (SOD) that converts the superoxide into hydrogen peroxide was observed in core members except for CFX14 and ATM01. The superoxide reductase (SOR) can also detoxify ROS by catalyzing the reduction of superoxide, but it was only observed in the two anammox MAGs. The hydrogen peroxide from the activity of SODs and SORs is further detoxified by catalase into water and oxygen. Alternately, the peroxidase oxidizes a wide variety of organic and inorganic substrates to convert hydrogen peroxide to water (Nobrega and Pauleta, 2019). Catalase was only observed in four *Proteobacteria* MAGs. PRO04, PRO05, PLA01, PLA03, and KSB01 were observed to produce catalase-peroxidases. Periplasmic cytochrome *c* peroxidases mediate the removal of periplasmic hydrogen peroxide by converting it to water. They were observed in all anammox and nitrifying MAGs and some heterotrophic MAGs (CFX09, PLA01, and PLA03). Other types of peroxidases, including peroxiredoxin, thioredoxin-dependent peroxiredoxin, and glutathione peroxidase, were scattered in some MAGs.

Thioredoxin, thioredoxin reductase, and NADPH constitute the thioredoxin system, which repairs the oxidized proteins through the reduction of disulfide bridges (Ezraty et al., 2017). Thioredoxin and thioredoxin reductase were the most prevalent antioxidant enzymes occurring in all core MAGs. Glutaredoxins, which provide complementary effects for the thioredoxin functions (Lu and Holmgren, 2014), were only observed in four *Proteobacteria* MAGs and NTP02. Methionine sulfoxide reductases (MsrA and MsrB) are another type of antioxidant enzyme that repairs methionine sulfoxides back to methionine (Ezraty et al., 2017). Genes encoding Msr were also prevalent in the core MAGs with the exception of PLA02, PLA03, CFX01, and CFX14. Both MsrA and MsrB were required for the full repair of oxidized Met residues because of their strict stereospecificity of substrates (Ezraty et al., 2017). The predominant heterotrophic CFX01 (and CFX14) may be more adaptive in the absence of oxygen because they only have SOD and thioredoxin systems.

### 3.6. Temporal dynamics of MAGs harboring *amo*

**Figure 5:**
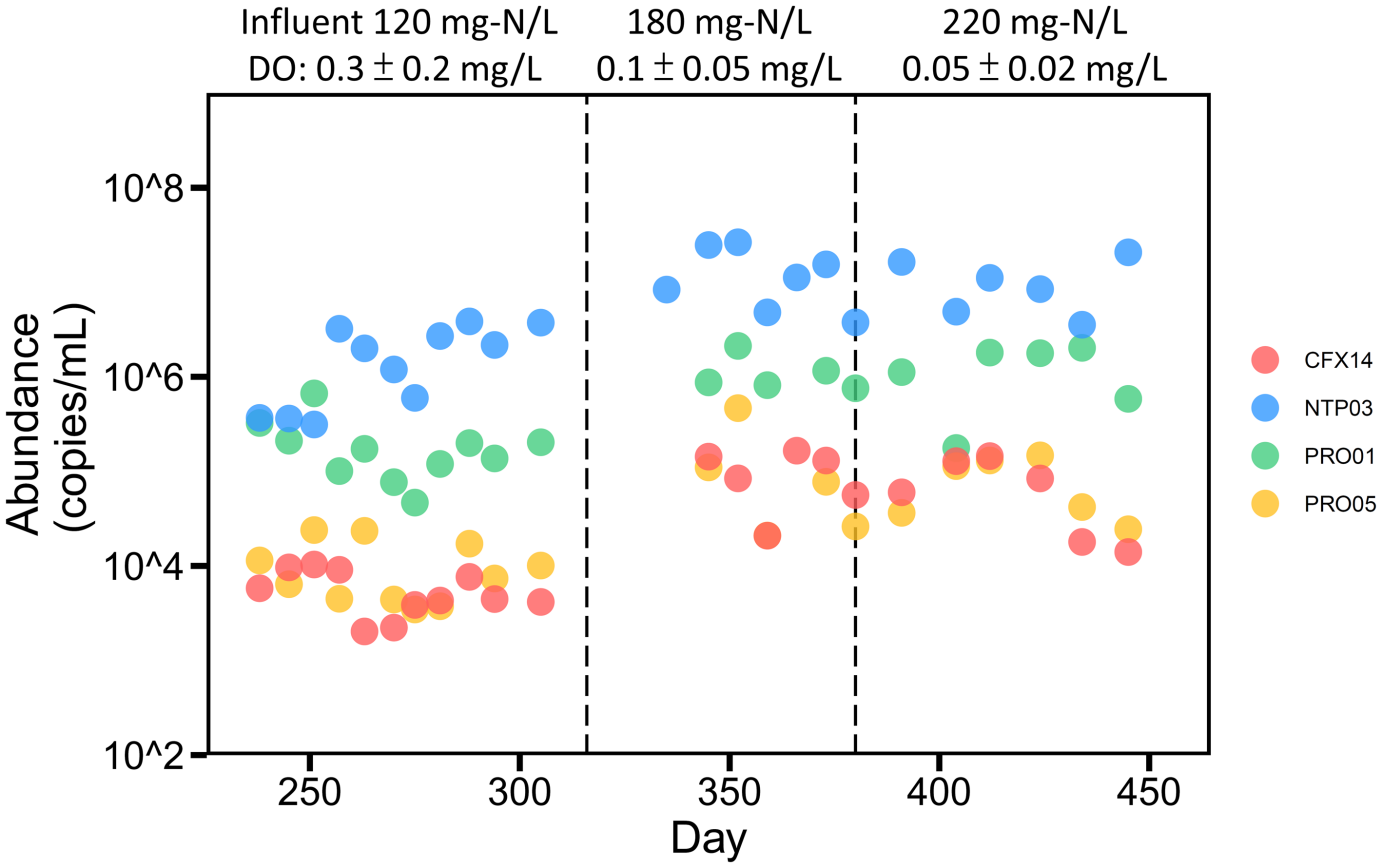
Temporal dynamics of potential ammonia-oxidizing members in the reactor. The reactor was fed with stepwise increased ammonium and operated under a constant retention time of 6.25 days. Flowrate of air supply was maintained at 9.7 ml/min on average. Aeration time stepwise decreased from 720 min/day (day 237–257), 480 (day 258–261), 120 (day 262–269) to 80 (since day 270).

Specific primer sets were designed (Supplementary Table S7) to reveal how the temporal dynamics of ammonia oxidizers were affected by the operation of the PN-A reactor. The influent ammonium concentration increased from 120 to 220 mg-N/L (Figure 5). When a constant aeration flowrate was maintained, the aeration time decreased from 720 to 120 min/day and then, from day 270, remained at 80 min/day. Further details regarding reactor operations and nitrogen removal performance are reported in our previous study (Shao and Wu, 2021). The qPCR analysis indicated that the *Nitrosomonas* PRO01 and comammox *Nitrospira* NTP03 were dominant at the beginning with comparable abundance (approximately 10^5^ copies/mL) and that comammox *Nitrospira* NTP03 outnumbered other ammonia oxidizers after aeration was decreased (Figure 5). As the input ammonium concentration increased, the levels of all ammonia oxidizers increased but at different rates.

We then compared the fold-changes for the average abundance of each ammonia oxidizer in the two periods, which were from days 237 to 257 with the highest air supply (DO = 0.51 ± 0.30 mg/l) and days 270 to 315 with the lowest air supply (DO = 0.16 ± 0.08 mg/l) under the same ammonium loadings. Only the abundance of comammox *Nitrospira* NTP03 increased (2.2 fold) as DO decreased, and the abundance of other ammonia oxidizers decreased. These results were consistent with the hypothesis that multiple copies of gene-encoding *bd*-type terminal oxidases favor the growth of comammox *Nitrospira* NTP03 under lower DO conditions. Moreover, the correlation analysis indicated that the abundance of ammonia oxidizers possessing high-affinity terminal oxidases exhibited a higher negative coefficient with aeration time (Supplementary Table S8). In particular, the temporal dynamics of comammox *Nitrospira* NTP03 exhibited the strongest negative correlation with aeration time among all ammonia-oxidizing members (Spearman’s coefficient of −0.63, *p* = 0.0009).

## 4. Discussion

### 4.1. Adaptation of anammox bacteria to the PN-A reactor

The genomic analysis conducted in this and previous studies (Ji et al., 2019; Yan et al., 2020) revealed the ability of anammox bacteria to relieve oxidative stress due to microaeration, as *Ca.* Brocadia and *Ca.* Jettenia both possessed a full capability to detoxify superoxide through H_2_O_2_ to H_2_O and to repair the oxidized proteins. The antioxidant capability is needed to support the activities of anammox bacteria in a microaerobic environment like the PN-A-reactor, although anammox bacteria were considered anaerobic. Our study further suggested that *Ca.* Brocadia AMX02 might be more capable of tolerating oxygen than *Ca.* Jettenia AMX01, because the AMX02 additioanlly possessed a class II RNR and a complete set of genes for methionine sulfoxide reductases. This finding reveals that the oxygen tolerance of anammox bacteria is species-dependent (Oshiki et al., 2016), which may lead to the oxygen-driven niche partition.

Nitrogen loading could be another determining factor for niche partitioning of anammox genera (Oshiki et al., 2016). The predominant anammox species was *Ca.* Jettenia AMX01 rather than *Ca.* Brocadia species as reported in previous studies where the anammox-associated reactors used higher ammonia loadings than this study (>10 times, Table 1). This is consistent with the fact that *Ca.* Jettenia has a lower requisite maintenance energy than *Ca.* Brocadia (Zhang et al., 2017; Zhang and Okabe, 2020) and were thus more dominant at low nitrogen loadings (<0.02 kg-N/m^3^/day). In our previous study, *Ca.* Brocadia outcompeted *Ca.* Jettenia at the loading of >0.035 kg-N/m^3^/day (Shao and Wu, 2021), suggesting niche differentiation by nitrogen loading.

**Table 1:** Influential and dominant MAGs from metagenomic analyzes of anammox and single-stage PN-A reactors.

Process	Reactor type	Nitrogen loading (kg-N/m^3^/day)	Influent			Controlled DO (mg/L)	Predominant microbial populations		References
			Ammonium (mg-N/L)	Nitrite (mg-N/L)	COD (mg/L)		Anammox genus	Heterotrophic phylum	
AMX	SBR	0.443	462	424	405	Anaerobic	*Ca.* Brocadia	Bacteroidota (Chlorobi)	Lawson et al. (2017)
AMX	SBR	0.03–0.7	50–250	50–320	Inorganic	Anaerobic	*Ca.* Brocadia^a^	Armatimonadota, Bacteroidota (Chlorobi), Chloroflexota, Alpha Proteobacteria^a^	Zhao et al. (2018)
AMX	CSTR	1.23	160	240	Inorganic	0–0.4	*Ca.* Brocadia	Proteobacteria (family Pasteurellaceae)	Ji et al. (2019, 2021)
PN-A	Granular anammox reactor	> 1^b^	266.8	N.A.^c^	197	0–3	*Ca.* Brocadia	Bacteroidota (Chlorobi)	Speth et al. (2016)
PN-A	SBR	0.375	250	0	Inorganic	0.1–0.8	*Ca.* Brocadia	Armatimonadota, Planctomycetota, Chloroflexota	Wang et al. (2019)
PN-A	CSTR	0.0192	120	0	Inorganic	0.1–0.3	*Ca.* Jettenia	Chloroflexota	This study

a Dominated at stage with the highest nitrogen loading.

b The nitrogen loading was reported in a previous study.

c Not available.

### 4.2. Adaptation of diverse ammonia-oxidizing bacteria to the PN-A reactor

Diverse core members that potentially oxidize ammonia to nitrite, including *Nitrosomonas*, comammox *Nitrospira,* and a *Chloroflexota*-like species, were observed in this study, suggesting the ammonia-oxidizing functional redundancy in association with low ammonia loadings. Although *Nitrosomonas* was a prevalent AOB in the PN-A systems (Speth et al., 2016; Wang et al., 2019), comammox *Nitrospira* NTP03 was more abundant than other AOB in our reactor, revealing the growth superiority in the reactor environment of this study. In addition to the high affinity to ammonium (Kits et al., 2017; Sakoula et al., 2021), comammox *Nitrospira* NTP03 possesses the microaerophilic-related *r*TCA pathway for carbon fixation (Lucker et al., 2010; Palomo et al., 2018) and high oxygen-affinity *bd*-type terminal oxidase genes (*K_m_* ranging from 3 to 300 nM) (Canfield and Kraft, 2022). The kinetic and genomic features account for the predominance of comammox *Nitrospira* species in the microaerobic environment.

*Chloroflexota* CFX14, which harbored *amoABC* and *hao* genes for ammonia oxidation, is a novel ammonia oxidizer and could be more adaptable to the low-DO environments in light of the ability to encode *bd*-type high oxygen-affinity terminal oxidases. Sorokin et al. (2012) reported the first nitrite-oxidizing strain, *Nitrolancetus hollandicus*, in the phylum *Chloroflexota*. However, no corresponding member harboring *amo* has been reported to date. The *amo* sequence of CFX14 was highly similar to the counterpart of *Nitrosospira in* the phylum *Proteobacteria*, but the overall genomic contents were distantly related to the phylum *Chloroflexota*. These discrepancies between gene phylogeny and species phylogeny may have resulted from horizontal gene transfer. Similar to comammox *Nitrospira* NTP03, *Chloroflexota* CFX14 increased more than that of *Nitrosomonas*-related MAGs (commonly, *aa3*-type terminal oxidase with *K_m_* ranging from 250 to 4,300 nM) as the air supply to the reactor decreased (Figure 5). However, *Chloroflexota* CFX14 did not reach as high an abundance as the comammox *Nitrospira* NTP03 in the studied reactor. Despite the unknown in the kinetics of ammonia utilization, the poor growth could be attributed to less potential for ROS defense. Interestingly, although *Nitrosomonas* PRO05 possessed an additional copy of the high oxygen-affinity terminal oxidase (*cbb3*-type with *K_m_* ranging from 7 to 250 nM) gene (Canfield and Kraft, 2022), its abundance was constantly lower than the abundance of the other species, PRO01, suggesting that other factors, such as ammonia oxidation kinetics, may aid the residence of *Nitrosomonas* species in low DO environments (Park and Noguera, 2007).

### 4.3. Adaptation of heterotrophic bacteria to the PN-A reactor

*Chloroflexota* CFX01 was the most abundant heterotrophic species in this study. This is different from previous studies, in which the *Chlorobi* species was highly dominant in anammox and in PN-A reactors that received real wastewater containing ammonium and COD (Speth et al., 2016; Lawson et al., 2017), and indicates that the predominance of heterotrophic taxa could be associated with the availability of exogenous and endogenous organic matters. Nitrogen loadings could also affect the dominance of heterotrophic taxa. Zhao et al. (2018) found that bacterial species related to *Chloroflexota* and *Armatimonadota* dominated the PN-A consortia with low (0.03–0.05 kg-N/m^3^/day) and high nitrogen loadings (0.15–0.7 kg-N/m^3^/day), respectively. Collectively, it is suggested a niche of *Chloroflexota* species in the PN-A reactor treating inorganic wastewater at low nitrogen loadings.

Notably, the success of heterotrophic CFX01 in the reactor of this study might benefit from the interactions with co-existing microbes *via* exchanges of endogenous substrates (EPS and nitrate) and specific growth factors (vitamins B6 and B12), as well as the collective mitigation of oxidative stress. The *Chloroflexota* CFX01 genome is characterized by poor H_2_O_2_ detoxification (Figure 4). The *bd*-type terminal oxidase, which has proposed a similar activity to catalase or peroxidase in *Escherichia coli* (Borisov et al., 2013; Al-Attar et al., 2016), might self-implement H_2_O_2_ detoxification. The antioxidant activities, including ROS detoxifying enzymes and O_2_-scavenging *bd*- and *cbb_3_*-type terminal oxidases (Borisov et al., 2021) provided by the co-existing members may also be helpful for ROS defense–deficient microbes like CFX01 and CFX14 (Kim et al., 2021). The antioxidant-oriented dependency between species has been described for AOA that commonly lack the genes encoding H_2_O_2_-detoxifying enzymes (Kim et al., 2016); thus, they rely highly on co-existing microbes for H_2_O_2_ detoxification (Bayer et al., 2019). Moreover, *Chloroflexota* CFX01 requires vitamin B12 for DNA synthesis but lacks a corresponding biosynthesis pathway. However, it can encode corresponding ABC transporters (Supplementary Data 2) to uptake cobalamin from suppliers such as anammox bacteria and *Nitrospira.* As exemplified with vitamin B6, autotrophic bacteria in this study could benefit from cross-feeding with co-existing heterotrophs, including the high-abundance CFX01. As observed previously (Wang et al., 2019) and in this study, the biosynthesis pathway of methionine was incomplete in anammox bacteria (AMX01 and AMX02). Thus, the growth may benefit from the exocellular supply of methionine from *Chloroflexota* CXF01 (and CFX09), where a nearly complete pathway of methionine synthesis was detected.

In this study, heterotrophic MAGs commonly encoded oxygen-independent class II RNRs, and terminal oxidases of both high- (*bd*- or *cbb_3_*-type) and low-affinity (*aa_3_* type), highlighting their ecological versatility in the alternate transition from microaerobic to anoxic environments. Because the antioxidant enzymes may differ in their affinities to the ROS species (Seaver Lauren and Imlay James, 2001; Singh et al., 2008). Whether predicted antioxidant enzymes have different affinities to the targeting ROS species remains largely unknown and requires further investigation.

### 4.4. Application implications

Currently, it is challenging to apply the PN-A method to low-strength mainstream wastewater, since the free-ammonia inhibition strategy may not effectively suppress the NOB activity (Wang et al., 2022). Thus, additional strategies, such as limiting oxygen supply with intermittent microaeration, would be needed (Wang et al., 2022). Because of the excellent ability in anoxic growth with a high ammonia affinity, the comammox *Nitrospira* represents an ideal ammonia oxidizer to co-work with anammox bacteria for converting ammonia nitrogen to dinitrogen in PN-A in the context of low DO and ammonium (Shao and Wu, 2021). As the characteristics of little N_2_O production and low aeration energy, the comammox *Nitrospira* as a dominant ammonia oxidizer in the PN-A can be an attractive method for ammonium removal in the emerging net-zero emission era. More studies on how to regulate ammonia- and nitrite-oxidizing activities of comammox *Nitrospira* in an anoxic environment are needed in the future.

Despite little contribution to the nitrogen loss in this study, the facultative heterotrophs can play a more crucial role in PN-A reactor when applied to the mainstream in the presence of COD concentrations but still within a proper C/N (~1–2.5) (Lotti et al., 2015; Kouba et al., 2016; Li et al., 2017). More oxygen supply could be needed for COD but to maintain similar microaerobic-anoxic conditions of this study for PN-A activities. In this regard, the facultative heterotrophs can facilitate optimal nitrogen removals with the cooperation of AOB, comammox *Nitrospira*, and anammox bacteria. Since the consortium members possess various oxygen affinities and interact closely regarding the metabolisms related to nitrogen, carbon, and nutrients, as well as oxygen, in-depth research on microbial composition dynamics, functions, and interactions under intermittent microaeration is required to provide a generalized framework for optimizing mainstream PN-A applications.

## 5. Conclusion

Our study provided insight into the genomics and community ecology of core microbes under low DO conditions in PN-A reactors receiving a low ammonium load; the novel findings could facilitate the precise management of PN-A systems for low ammonia-related wastewater.

## Data availability statement

Metagenomic sequence data have been deposited in the NCBI under BioProject PRJNA862642. Metagenomic sequences are available in the Sequence Read Archive repository under the accession numbers SRR20682006 and SRR20682005, and 18 core MAGs are available under the BioSample accession numbers SAMN30011305, SAMN30011306, and SAMN29987002 to SAMN29987019.

## Author contributions

J-HW conceived the study. J-HW and Y-HS designed the analysis. Y-HS collected and processed the sludge samples, performed experiments and reactor operation, and drafted the manuscript. Y-HS, Y-WW, and MN conducted the data analysis. J-HW and Y-WW revised the manuscript and edited the final version. All authors helped edit the manuscript. All authors read and approved the final manuscript.

## Funding

This study was supported by the Ministry of Science and Technology, Taiwan (grant number 109-2221-E-006 -093-MY3). The open access publication fee was supported in part by Higher Education Sprout Project, Ministry of Education to the Headquarters of University Advancement at National Cheng Kung University. The granting agencies have no role in this study.

## Conflict of interest

The authors declare that the research was conducted in the absence of any commercial or financial relationships that could be construed as a potential conflict of interest.

## Publisher’s note

All claims expressed in this article are solely those of the authors and do not necessarily represent those of their affiliated organizations, or those of the publisher, the editors and the reviewers. Any product that may be evaluated in this article, or claim that may be made by its manufacturer, is not guaranteed or endorsed by the publisher.

## Footnotes

^1^ https://sourceforge.net/projects/bbmap/
