# Supplementary material for: Genome-centered metagenomics illuminates adaptations of core members to a partial Nitritation–Anammox bioreactor under periodic microaeration
Source: Front Microbiol. 2023 Jan 26;14:1046769. doi: 10.3389/fmicb.2023.1046769 (PMC9909701; doi:10.3389/fmicb.2023.1046769)
Supplement: Supplementary file 1 [file Table_1.DOCX]

**Supplementary Information**

**Genome-Centered Metagenomics Illuminates Adaptations of Core Members to a Partial Nitritation–Anammox Bioreactor under Periodic Microaeration**

Yung-Hsien Shao^1^, Yu-Wei Wu^2^, Muhammad Naufal^1^, and Jer-Horng Wu^1^*

^1^Department of Environmental Engineering, National Cheng Kung University, Taiwan.

^2^Graduate Institute of Biomedical Informatics, College of Medical Science and Technology, Taipei Medical University, Taiwan.

*Corresponding author

E-mail: enewujh@ncku.edu.tw

Postal address: No.1, University Road, East District, Tainan City 701, Taiwan

**Supplementary methods**

**1. Processing of amplicon sequencing data**

To analyze the 16S rRNA gene sequences, the PCR-generated amplicons of V3-V4 hypervariable region of bacterial 16S rRNA gene were sequenced using the Illumina MiSeq platform. The obtained sequences were processed using the DADA2 pipeline (Callahan et al. 2016) with the q2-dada2 plugin in QIIME 2 (v2019.4) (Bolyen et al. 2019). Briefly, the raw sequence data were demultiplexed and trimmed and then further processed for merging paired-end reads, removing chimeras, and generating amplicon sequence variants (ASVs). Taxonomy was assigned to ASVs with a Scikit-learn naïve Bayesian classifier trained on the SILVA 128 rRNA database (Quast et al. 2013) using the q2-feature-classifier plugin with default settings (Bokulich et al. 2018).

**2. Primer design and validation**

The oligonucleotide primers targeting the *amo* gene of specific MAGs were designed using Primer-BLAST (Ye et al. 2012) based on RefSeq Representative Genome Database with default settings of primer specificity stringency. Primer pairs fulfilled the following requirements were selected: (i) primer length of 18-23 bp, (ii) amplicon length of 100-300 bp, (iii) primer GC content of 50-60%, (vi) melting temperature (T_m_) difference between reverse and forward primers < 5 °C and, (v) no repeats of Gs or Cs longer than three bases. Primer pairs that met the above criteria were then selected for Primer-BLAST runs against the nr database to ensure specificity to our target gene.

**Table S1.** Relative abundance of AOB, NOB and anammox bacteria detected by using 16S rDNA amplicon sequencing and read-based metagenomics.

| Day | Method | Relative abundance (%) | | | | |
| --- | --- | --- | --- | --- | --- | --- |
|  |  | *Ca*. Jettenia | *Ca*. Kuenenia | *Ca*. Brocadia | *Nitrospira* | *Nitrosomonas* |
| 191 | 16S sequencing | 32.15 | 0.15 | N.D. | 7.11 | 14.97 |
|  | Metagenomics* | 11.35 | 0.03 | 0.42 | 7.68 | 2.40 |
| 235 | 16S sequencing | 20.45 | 0.35 | N.D. | 7.39 | 16.91 |
|  | Metagenomics** | 8.44 | 0.02 | 0.32 | 7.97 | 1.84 |

*67.6% of clean reads could not be assigned to a genus

**71.3% of clean reads could not be assigned to a genus

**Table S2.** Genomic features of 18 core MAGs in this study

|  | Bin ID | Comp.  (%)* | Cont.  (%)** | GC content | Genome size (bp) | # scaffolds | scaffolds N50 | Taxonomy | Closest placement reference  (ANI %, accession number) |
| --- | --- | --- | --- | --- | --- | --- | --- | --- | --- |
| 1 | CFX01 | 99.55 | 3.36 | 0.56 | 6116110 | 194 | 129721 | Bacteria; Chloroflexota; Anaerolineae; SBR1031; UBA2796; UBA2796 | Anaerolineales bacterium UBA2796 (76.8, GCA_002352035.1) |
| 2 | CFX09 | 98.28 | 8.62 | 0.54 | 3799100 | 331 | 23200 | Bacteria; Chloroflexota; Anaerolineae; Anaerolineales; EnvOPS12; OLB14; OLB14 sp008363285 | Chloroflexi bacterium (98.6, GCA_008363285.1) |
| 3 | CFX14 | 75.81 | 5.45 | 0.59 | 3792667 | 805 | 9705 | Bacteria; Chloroflexota; Anaerolineae; SBR1031; UBA3940; ; | N/A |
| 4 | AMX01 | 95.60 | 1.65 | 0.39 | 3671969 | 72 | 100945 | Bacteria; Planctomycetota; Brocadiae; Brocadiales; Brocadiaceae; Jettenia | Ca. Jettenia caeni (94.5, GCF_000296795.1) |
| 5 | AMX02 | 100.00 | 3.85 | 0.42 | 3278046 | 189 | 45851 | Bacteria; Planctomycetota; Brocadiae; Brocadiales; Brocadiaceae; Brocadia; Brocadia sapporoensis | Ca. Brocadia sapporoensis (96.5, GCF_001753675.2) |
| 6 | NTP02 | 97.67 | 2.73 | 0.60 | 4534387 | 222 | 63336 | Bacteria; Nitrospirota; Nitrospiria; Nitrospirales; Nitrospiraceae; Nitrospira_A; Nitrospira_A sp003456605 | Nitrospira defluvii (99.4, GCA_011525625.1) |
| 7 | NTP03 | 96.70 | 3.64 | 0.57 | 5526973 | 509 | 30167 | Bacteria; Nitrospirota; Nitrospiria; Nitrospirales; Nitrospiraceae; Nitrospira_F; | N/A |
| 8 | NTP04 | 96.82 | 5.51 | 0.61 | 4534672 | 111 | 87392 | Bacteria; Nitrospirota; Nitrospiria; Nitrospirales; Nitrospiraceae; ; | N/A |
| 9 | NTP05 | 96.76 | 5.86 | 0.60 | 4313236 | 79 | 163650 | Bacteria; Nitrospirota; Nitrospiria; Nitrospirales; Nitrospiraceae; Nitrospira_A; Nitrospira_A sp001567445 | Nitrospira sp. OLB3 (99.8, GCA_001567445.1) |
| 10 | PRO01 | 99.52 | 0.98 | 0.49 | 3121699 | 86 | 102978 | Bacteria; Proteobacteria; Gammaproteobacteria; Burkholderiales; Nitrosomonadaceae; Nitrosomonas | Proteobacteria bacterium SG_bin4 (86.3, GCA_002083395.1) |
| 11 | PRO02 | 92.05 | 2.13 | 0.67 | 3284777 | 162 | 65981 | Bacteria; Proteobacteria; Gammaproteobacteria; Burkholderiales; Rhodocyclaceae; UTPRO2; UTPRO2 sp008933825 | Rhodocyclaceae bacterium (98.5, GCA_008933825.1) |
| 12 | PRO04 | 97.80 | 0.00 | 0.66 | 2711748 | 46 | 352277 | Bacteria; Proteobacteria; Gammaproteobacteria; UBA5335; UBA5335; Macondimonas; | Proteobacteria bacterium (88.7, GCA_002862965.1) |
| 13 | PRO05 | 98.55 | 0.62 | 0.51 | 2827150 | 99 | 64829 | Bacteria; Proteobacteria; Gammaproteobacteria; Burkholderiales; Nitrosomonadaceae; Nitrosomonas; Nitrosomonas europaea | Nitrosomonas europaea ATCC 25978 (98.8, GCF_900167395.1) |
| 14 | PLA01 | 96.59 | 0.00 | 0.63 | 4430612 | 72 | 249119 | Bacteria; Planctomycetota; Phycisphaerae; UBA1845; UTPLA1; PLA3 | Phycisphaerae bacterium RAS2 (88.4, GCF_007753915.1) |
| 15 | PLA02 | 96.52 | 0.00 | 0.70 | 3683191 | 60 | 142871 | Bacteria; Planctomycetota; Phycisphaerae; Phycisphaerales; SM1A02; CAADGN01; CAADGN01 sp014584315 | Planctomycetes bacterium (99.6, GCA_014584315.1) |
| 16 | PLA03 | 91.98 | 4.55 | 0.64 | 3880475 | 196 | 44575 | Bacteria; Planctomycetota; UBA8742; UBA2392; UBA2392; UBA2392; UBA2392 sp002343805 | Planctomycetes bacterium UBA2392 (99.0, GCA_002343805.1) |
| 17 | KSB01 | 95.54 | 2.20 | 0.53 | 7192567 | 267 | 65326 | Bacteria; KSB1; UBA2214; DRLW01; QEVD01; QEVD01; | candidate division KSB1 bacterium (95, GCA_003576975.1) |
| 18 | ATM01 | 90.28 | 0.93 | 0.61 | 2628781 | 169 | 34765 | Bacteria; Armatimonadota; Fimbriimonadia; Fimbriimonadales; Fimbriimonadaceae; OLB18; OLB18 sp001567425 | Armatimonadetes bacterium OLB18 (98.0, GCA_001567425.1) |

**Table S3.** List of selected KO in the analysis of nitrogen, carbon and oxygen metabolism

| Name | k number | Definition |
| --- | --- | --- |
| Nitrogen-related metabolism | | |
| pmoA/amoA | K10944 | methane/ammonia monooxygenase subunit A |
| pmoB/amoB | K10945 | methane/ammonia monooxygenase subunit B |
| pmoC/amoC | K10946 | methane/ammonia monooxygenase subunit C |
| hao | K10535 | hydroxylamine dehydrogenase |
| narG/nxrA | K00370 | nitrate reductase / nitrite oxidoreductase, alpha subunit |
| narH/nxrB | K00371 | nitrate reductase / nitrite oxidoreductase, beta subunit |
| narI | K00374 | nitrate reductase gamma subunit |
| napA | K02567 | nitrate reductase (cytochrome) |
| napB | K02568 | nitrate reductase (cytochrome), electron transfer subunit |
| nirS | K15864 | nitrite reductase (NO-forming) |
| nirK | K00368 | nitrite reductase (NO-forming) |
| norB | K04561 | nitric oxide reductase subunit B |
| norC | K02305 | nitric oxide reductase subunit C |
| nosZ | K00376 | nitrous-oxide reductase |
| nirB | K00362 | nitrite reductase (NADH) large subunit |
| nirD | K00363 | nitrite reductase (NADH) small subunit |
| nrfA | K03385 | nitrite reductase (cytochrome c-552) |
| nrfH | K15876 | cytochrome c nitrite reductase small subunit |
| hzs | K20932 | hydrazine synthase subunit |
| hzs | K20933 | hydrazine synthase subunit |
| hzs | K20934 | hydrazine synthase subunit |
| hdh | K20935 | hydrazine dehydrogenase |
| narB | K00367 | ferredoxin-nitrate reductase |
| nasA | K00372 | assimilatory nitrate reductase catalytic subunit |
| nasB | K00360 | assimilatory nitrate reductase electron transfer subunit |
| NR | K10534 | nitrate reductase |
| nirA | K00366 | ferredoxin-nitrite reductase |
| NIT-6 | K17877 | nitrite reductase |
| ncd2/npd | K00459 | nitronate monooxygenase |
| formamidase | K01455 | formamidase |
| nitrilase | K01501 | nitrilase |
| cynS | K01725 | cyanate lyase |
| NAO | K19823 | nitroalkane oxidase |
| amiE | K01426 | amidase |
| ureC | K01428 | urease subunit alpha |
| ureB | K01429 | urease subunit beta |
| ureA | K01430 | urease subunit gamma |
| amt | K03320 | ammonium transporter, Amt family |
| Rh | K06580 | ammonium transporter Rh |
| yfdC | K21990 | formate-nitrite transporter family protein |
| nirC | K02598 | nitrite transporter |
| narK | K02575 | nitrate/nitrite transporter |
| Carbon-related metabolism | | |
| aclA | K15230 | ATP-citrate lyase alpha-subunit |
| aclB | K15231 | ATP-citrate lyase beta-subunit |
| cbbL | K01601 | ribulose-bisphosphate carboxylase large chain |
| cbbS | K01602 | ribulose-bisphosphate carboxylase small chain |
| acsA | K00198 | anaerobic carbon-monoxide dehydrogenase catalytic subunit |
| acsB | K14138 | acetyl-CoA synthase |
| mcl | K08691 | malyl-CoA/(S)-citramalyl-CoA lyase |
| mct | K14470 | 2-methylfumaryl-CoA isomerase |
| mch | K14449 | 2-methylfumaryl-CoA hydratase |
| meh | K09709 | 3-methylfumaryl-CoA hydratase |
| mcr | K14468 | malonyl-CoA reductase / 3-hydroxypropionate dehydrogenase (NADP+) |
| poxB | K00156 | pyruvate dehydrogenase (quinone) |
| pta | K13788 | phosphate acetyltransferase |
| pta | K00625 | phosphate acetyltransferase |
| ackA | K00925 | acetate kinase |
| acyP | K01512 | acylphosphatase |
| pflD | K00656 | formate C-acetyltransferase |
| ldh | K00016 | L-lactate dehydrogenase |
| adhE | K04072 | acetaldehyde dehydrogenase |
| acdAB | K24012 | acetate---CoA ligase (ADP-forming) |
| acs | K01895 | acetyl-CoA synthetase |
| Ribonucleoside reductase | | |
| nrdA | K00525 | ribonucleoside-diphosphate reductase alpha chain |
| nrdJ | K00524 | ribonucleotide reductase, class II |
| nrdD | K21636 | ribonucleoside-triphosphate reductase |
| Oxygen-related metabolism | | |
| cyoB | K02298 | cytochrome o ubiquinol oxidase subunit I |
| qoxB | K02827 | cytochrome aa3-600 menaquinol oxidase subunit I |
| coxA, ctaD | K02274 | cytochrome c oxidase subunit I (HCO class A) |
|  | K02274 | cytochrome c oxidase subunit I (HCO class B/C) |
| cydA | K00425 | cytochrome bd ubiquinol oxidase subunit I |
| ccoN | K00404 | cytochrome c oxidase cbb3-type subunit I |
| trxB, TRR | K00384 | thioredoxin reductase (NADPH) |
| trxA | K03671 | thioredoxin 1 |
| trxC | K03672 | thioredoxin 2 |
| SOD2 | K04564 | superoxide dismutase, Fe-Mn family |
| SOD1 | K04565 | superoxide dismutase, Cu-Zn family |
| sodN | K00518 | nickel superoxide dismutase |
| katE, CAT, catB, srpA | K03781 | catalase |
|  | K07217 | Mn-containing catalase |
| katG | K03782 | catalase-peroxidase |
| PRDX2_4, ahpC | K03386 | peroxiredoxin 2/4 |
| gpx, btuE, bsaA | K00432 | glutathione peroxidase |
| tpx | K11065 | thioredoxin-dependent peroxiredoxin |
|  | K00428 | cytochrome c peroxidase |
| AHP1 | K14171 | alkyl hydroperoxide reductase 1 |
| npr | K05910 | NADH peroxidase |
|  | K00430 | peroxidase |
| PRDX6 | K11188 | peroxiredoxin 6 |
| PRDX5 | K11187 | peroxiredoxin 5 |
| PRDX1 | K13279 | peroxiredoxin 1 |
| PRDX3 | K20011 | peroxiredoxin 3 |
| dfx | K05919 | superoxide reductase |
| fprB | K19824 | rubrerythrin |
| grxC, GLRX, GLRX2 | K03676 | glutaredoxin 3 |
| grxD, GLRX5 | K07390 | monothiol glutaredoxin |
| grxA | K03674 | glutaredoxin 1 |
| grxB | K03675 | glutaredoxin 2 |
| msrA | K07304 | methionine sulfoxide reductase A |
| msrB | K07305 | methionine sulfoxide reductase B |

**Table S4.** Genomic features of *Nitrospira* MAGs

| Genome | Completeness | Contamination | G+C content | Size | Accession number | AAI with CB_NTP03 |
| --- | --- | --- | --- | --- | --- | --- |
| Ca.Nitrospira_kreftii_isolate_comreactor17 | 96.76 | 2.78 | 54.47 | 4.13 | CP047423 | **79.87** |
| Ca.Nitrospira_nitrificans_COMA2 | 96.76 | 2.73 | 56.59 | 4.12 | GCF_001458775 | 75.74 |
| Ca.Nitrospira_nitrosa_COMA1 | 96.76 | 2.27 | 54.80 | 4.42 | GCF_001458735 | 74.61 |
| CB_NTP03 | 96.70 | 3.64 | 56.60 | 5.53 |  | 100 |
| Nitrospira_defluvii | 96.82 | 6.77 | 58.92 | 4.84 | GCF_905220995 | 63.88 |
| Nitrospira_inopinata_ENR4 | 96.82 | 4.77 | 59.23 | 3.30 | GCF_001458695 | 69.69 |
| Nitrospira_japonica | 96.82 | 3.92 | 58.96 | 4.08 | GCF_900169565 | 64.66 |
| Nitrospira_lenta | 95.85 | 3.18 | 57.88 | 3.76 | GCF_900403705 | 67.65 |
| Nitrospira_moscoviensis | 95.91 | 6.55 | 61.99 | 4.59 | GCF_001273775 | 69.19 |
| Nitrospira_sp.AMP-bin1 | 96.76 | 1.82 | 54.63 | 4.36 | GCA_018242825 | 73.75 |
| Nitrospira_sp.CTRL-LIN-TMP-bin1 | 95.85 | 3.23 | 55.38 | 4.21 | GCA_018242685 | 75.36 |
| Nitrospira_sp.KAN-bin1 | 96.76 | 1.82 | 54.79 | 4.68 | GCA_018242765 | 73.42 |
| Nitrospira_sp.KAN-bin2 | 95.85 | 1.82 | 54.59 | 4.02 | GCA_018242725 | 74.19 |
| Nitrospira_sp.KM1 | 95.91 | 2.73 | 55.98 | 4.51 | GCF_011405515 | 64.14 |
| Nitrospira_sp.ND1 | 97.67 | 2.73 | 58.87 | 4.45 | GCF_900170025 | 63.21 |
| Nitrospira_sp.palsa_1310 | 96.76 | 3.18 | 56.94 | 4.34 | GCA_003135435 | 64.96 |
| Nitrospira_sp.RBC069 | 95.85 | 2.73 | 54.50 | 4.53 | GCA_902500775 | 73.38 |
| Nitrospira_sp.RSF1 | 95.83 | 3.01 | 55.24 | 3.73 | GCA_005116965 | 75.6 |
| Nitrospira_sp.RSF3 | 95.85 | 3.18 | 55.56 | 3.94 | GCA_005116835 | 64.35 |
| Nitrospira_sp.RSF7 | 95.85 | 2.73 | 56.19 | 2.63 | GCA_005116825 | 67.27 |
| Nitrospira_sp.RSF9 | 95.85 | 2.83 | 54.96 | 3.86 | GCA_005116745 | 75.61 |
| Nitrospira_sp.SG-bin1 | 95.85 | 3.69 | 56.08 | 4.42 | GCA_002083365 | 75.15 |
| Nitrospira_sp.SG-bin2 | 95.85 | 3.69 | 56.77 | 3.66 | GCA_002083405 | 70.69 |
| Nitrospira_sp.UW-LDO-01 | 95.80 | 3.64 | 54.93 | 3.91 | GCA_002254365 | 74.72 |
| Nitrospira_sp.YR-XLJ-3 | 95.17 | 3.69 | 57.24 | 2.77 | GCA_015903955 | 72.43 |
| Nitrospira_UBA5698 | 95.00 | 3.86 | 55.06 | 4.38 | GCA_002420115 | 74.5 |

**Table S5.** List of detected genera in order betaproteobacteriales using 16S rDNA amplicon sequencing in two sludge samples

| Taxonomy | Relative abundance in day 191 (%) | Relative abundance in day 235 (%) |
| --- | --- | --- |
| D_0__Bacteria;D_1__Proteobacteria;D_2__Gammaproteobacteria;D_3__Betaproteobacteriales;D_4__B1-7BS;Ambiguous_taxa | 0.08 | 0.38 |
| D_0__Bacteria;D_1__Proteobacteria;D_2__Gammaproteobacteria;D_3__Betaproteobacteriales;D_4__B1-7BS;__ | 0.27 | 0.28 |
| D_0__Bacteria;D_1__Proteobacteria;D_2__Gammaproteobacteria;D_3__Betaproteobacteriales;D_4__Burkholderiaceae;D_5__Alicycliphilus | 0.10 | 0.28 |
| D_0__Bacteria;D_1__Proteobacteria;D_2__Gammaproteobacteria;D_3__Betaproteobacteriales;D_4__Burkholderiaceae;D_5__Aquabacterium | 0.07 | 0.00 |
| D_0__Bacteria;D_1__Proteobacteria;D_2__Gammaproteobacteria;D_3__Betaproteobacteriales;D_4__Burkholderiaceae;D_5__Comamonas | 0.25 | 0.00 |
| D_0__Bacteria;D_1__Proteobacteria;D_2__Gammaproteobacteria;D_3__Betaproteobacteriales;D_4__Burkholderiaceae;D_5__Limnobacter | 0.16 | 0.27 |
| D_0__Bacteria;D_1__Proteobacteria;D_2__Gammaproteobacteria;D_3__Betaproteobacteriales;D_4__Burkholderiaceae;D_5__uncultured | 0.00 | 0.49 |
| D_0__Bacteria;D_1__Proteobacteria;D_2__Gammaproteobacteria;D_3__Betaproteobacteriales;D_4__Burkholderiaceae;__ | 0.85 | 1.18 |
| D_0__Bacteria;D_1__Proteobacteria;D_2__Gammaproteobacteria;D_3__Betaproteobacteriales;D_4__Methylophilaceae;D_5__UBA6140 | 0.09 | 0.09 |
| D_0__Bacteria;D_1__Proteobacteria;D_2__Gammaproteobacteria;D_3__Betaproteobacteriales;D_4__Nitrosomonadaceae;D_5__Nitrosomonas | 14.97 | 16.91 |
| D_0__Bacteria;D_1__Proteobacteria;D_2__Gammaproteobacteria;D_3__Betaproteobacteriales;D_4__Rhodocyclaceae;D_5__Denitratisoma | 4.01 | 4.99 |
| D_0__Bacteria;D_1__Proteobacteria;D_2__Gammaproteobacteria;D_3__Betaproteobacteriales;D_4__Rhodocyclaceae;D_5__Thauera | 1.73 | 3.99 |
| D_0__Bacteria;D_1__Proteobacteria;D_2__Gammaproteobacteria;D_3__Betaproteobacteriales;D_4__Rhodocyclaceae;D_5__uncultured | 0.78 | 1.10 |
| D_0__Bacteria;D_1__Proteobacteria;D_2__Gammaproteobacteria;D_3__Betaproteobacteriales;D_4__SC-I-84;__ | 0.14 | 0.98 |

**Table S6.** List of ORFs with inconsistent prediction results of types of terminal oxidase and ribonucleotide reductase using KEGG classification system and blastp search against nr database.

| Bin ID | ORF ID | KEGG annotation | Blastp annotation |
| --- | --- | --- | --- |
| Terminal oxidase | | | |
| CFX09 | JLAFPPAO_00962 | cytochrome c oxidase subunit I (K02274) | cbb3-type cytochrome c oxidase subunit I (MBE7434980.1) |
|  | JLAFPPAO_02886 | cytochrome c oxidase subunit I (K02274) | b(o/a)3-type cytochrome-c oxidase subunit 1 (KAA0270359.1) |
| PRO02 | FEBGKFLJ_02254 | cytochrome c oxidase subunit I (K02274) | cbb3-type cytochrome c oxidase subunit I (KAB2936211.1) |
|  | FEBGKFLJ_02511 | cytochrome c oxidase subunit I (K02274) | cbb3-type cytochrome c oxidase subunit I (MBE7420877.1) |
| PLA03 | POKBAJEO_00127 | cytochrome c oxidase cbb3-type subunit I (K00404) | Cytochrome c oxidase subunit 1, bacteroid (MBV6514506.1) |
|  | POKBAJEO_02516 | cytochrome c oxidase subunit I (K02274) | NUO15556.1 cbb3-type cytochrome c oxidase subunit I (NUO15556.1) |
| PRO05 | FPBBJOOK_00730 | cytochrome c oxidase subunit I (K02274) | cbb3-type cytochrome c oxidase subunit I (WP_011111304.1) |
| Ribonucleotide reductase | | | |
| CFX01 | MAPEBKBH_00478 | ribonucleoside-diphosphate reductase alpha chain (K00525) | Vitamin B12-dependent ribonucleoside-diphosphate reductase (CAG0935082) |
| NTP04 | CIFBLAND_04188 | ribonucleoside-diphosphate reductase alpha chain (K00525) | adenosylcobalamin-dependent ribonucleoside-diphosphate reductase (MBA5869405) |
|  | CIFBLAND_01597 | ribonucleoside-diphosphate reductase alpha chain (K00525) |  |
| PLA01 | ALMADPKO_02481 | ribonucleoside-diphosphate reductase alpha chain (K00525) | Ribonucleoside-diphosphate reductase NrdZ (QDV92455) |
|  | ALMADPKO_03566 | ribonucleoside-diphosphate reductase alpha chain (K00525) | adenosylcobalamin-dependent ribonucleoside-diphosphate reductase (MBA5867284) |
| NTP05 | DEEFEKBD_02559 | ribonucleoside-diphosphate reductase alpha chain (K00525) | ribonucleoside-diphosphate reductase, adenosylcobalamin-dependent (KXK07252) |
|  | DEEFEKBD_04046 | ribonucleoside-diphosphate reductase alpha chain (K00525) | adenosylcobalamin-dependent ribonucleoside-diphosphate reductase (WP_053379212) |
| NTP03 | ECDCFKHL_00651 | ribonucleoside-diphosphate reductase alpha chain (K00525) | vitamin B12-dependent ribonucleotide reductase (MBK9308424) |
|  | ECDCFKHL_03262 | ribonucleoside-diphosphate reductase alpha chain (K00525) | vitamin B12-dependent ribonucleotide reductase (MBS2022633) |
|  | ECDCFKHL_03850 | ribonucleoside-diphosphate reductase alpha chain (K00525) | vitamin B12-dependent ribonucleotide reductase (HDO52646) |
|  | ECDCFKHL_03999 | ribonucleoside-diphosphate reductase alpha chain (K00525) |  |
| CFX09 | JLAFPPAO_00415 | ribonucleoside-diphosphate reductase alpha chain (K00525) | adenosylcobalamin-dependent ribonucleoside-diphosphate reductase (KAA0276236) |
| PLA02 | IFCOPBLL_01514 | ribonucleoside-diphosphate reductase alpha chain (K00525) | hypothetical protein (MBC6954308) |
| PRO02 | FEBGKFLJ_02472 | ribonucleoside-diphosphate reductase alpha chain (K00525) |  |
| KSB01 | KGKHFNCM_04871 | ribonucleoside-diphosphate reductase alpha chain (K00525) | vitamin B12-dependent ribonucleotide reductase (NUM77462) |
| PLA03 | POKBAJEO_01733 | ribonucleoside-diphosphate reductase alpha chain (K00525) | hypothetical protein (MBV6514997) |
| ATM01 | ECNLFNEI_01793 | ribonucleoside-diphosphate reductase alpha chain (K00525) | vitamin B12-dependent ribonucleotide reductase (KAA0238671) |
| NTP02 | IBOJHJNA_00151 | ribonucleoside-diphosphate reductase alpha chain (K00525) | vitamin B12-dependent ribonucleotide reductase (WP_213041393) |
| AMX02 | HHEHPEGL_01319 | ribonucleoside-diphosphate reductase alpha chain (K00525) |  |

**Table S7.** Primers designed for detecting temporal dynamics of potential ammonia-oxidizing members

| Target gene | Primer | Sequence (5’ → 3’) | Primer length | Product length | Tm (℃) | GC content (%) |
| --- | --- | --- | --- | --- | --- | --- |
| PRO05 *amoC* | CB_PRO05_amoCF | TTCTATGGCTCATTCCCGGT | 20 | 269 | 58.5 | 50 |
|  | CB_PRO05_amoCR | ATGAGCTGTATGGCAAGACC | 20 |  | 57.7 | 50 |
| PRO01 *amoC* | CB_PRO01_amoCF | CAATTGGACAACCTGGACCCT | 21 | 223 | 60.2 | 52.4 |
|  | CB_PRO01_amoCR | AGGATGCTACACCGCATAC | 19 |  | 57.0 | 52.6 |
| NTP03 *amoA* | CB_NTP03_amoAF | ATGCATACCGCTCTCTTGTG | 20 | 177 | 58.1 | 50 |
|  | CB_NTP03_amoAR | TCCCAGCGCTAAGATACAGA | 20 |  | 57.9 | 50 |
| CFX14 *amoA* | CB_CFX14_amoAF | ACCACGGTAATTGCGTCCTT | 20 | 138 | 59.9 | 50 |
|  | CB_CFX14_amoAR | GTCGTTTATCATGCTCACTCGCC | 23 |  | 62.1 | 52.2 |

**Table S8.** Coefficient with significant *p* values (<0.05) are highlighted in bold.

| Operational parameter | Spearman’s correlation coefficient | | | |
| --- | --- | --- | --- | --- |
|  | PRO01 | PRO05 | CFX14 | NTP03 |
| pH | -0.13 | -0.21 | -0.15 | -0.27 |
| DO | -0.29 | -0.39 | -0.29 | -0.40 |
| Temperature | 0.07 | 0.20 | 0.21 | 0.08 |
| Aeration time | -0.21 | -0.29 | -0.33 | **-0.63** |
| Loading | **0.68** | **0.80** | **0.74** | **0.69** |

**Reference**

Bokulich, N.A., Kaehler, B.D., Rideout, J.R., Dillon, M., Bolyen, E., Knight, R., Huttley, G.A. and Gregory Caporaso, J. (2018) Optimizing taxonomic classification of marker-gene amplicon sequences with QIIME 2's q2-feature-classifier plugin. Microbiome 6(1), 90.

Bolyen, E., Rideout, J.R., Dillon, M.R., Bokulich, N.A., Abnet, C.C., Al-Ghalith, G.A., Alexander, H., Alm, E.J., Arumugam, M., Asnicar, F., Bai, Y., Bisanz, J.E., Bittinger, K., Brejnrod, A., Brislawn, C.J., Brown, C.T., Callahan, B.J., Caraballo-Rodriguez, A.M., Chase, J., Cope, E.K., Da Silva, R., Diener, C., Dorrestein, P.C., Douglas, G.M., Durall, D.M., Duvallet, C., Edwardson, C.F., Ernst, M., Estaki, M., Fouquier, J., Gauglitz, J.M., Gibbons, S.M., Gibson, D.L., Gonzalez, A., Gorlick, K., Guo, J., Hillmann, B., Holmes, S., Holste, H., Huttenhower, C., Huttley, G.A., Janssen, S., Jarmusch, A.K., Jiang, L., Kaehler, B.D., Kang, K.B., Keefe, C.R., Keim, P., Kelley, S.T., Knights, D., Koester, I., Kosciolek, T., Kreps, J., Langille, M.G.I., Lee, J., Ley, R., Liu, Y.X., Loftfield, E., Lozupone, C., Maher, M., Marotz, C., Martin, B.D., McDonald, D., McIver, L.J., Melnik, A.V., Metcalf, J.L., Morgan, S.C., Morton, J.T., Naimey, A.T., Navas-Molina, J.A., Nothias, L.F., Orchanian, S.B., Pearson, T., Peoples, S.L., Petras, D., Preuss, M.L., Pruesse, E., Rasmussen, L.B., Rivers, A., Robeson, M.S., 2nd, Rosenthal, P., Segata, N., Shaffer, M., Shiffer, A., Sinha, R., Song, S.J., Spear, J.R., Swafford, A.D., Thompson, L.R., Torres, P.J., Trinh, P., Tripathi, A., Turnbaugh, P.J., Ul-Hasan, S., van der Hooft, J.J.J., Vargas, F., Vazquez-Baeza, Y., Vogtmann, E., von Hippel, M., Walters, W., Wan, Y., Wang, M., Warren, J., Weber, K.C., Williamson, C.H.D., Willis, A.D., Xu, Z.Z., Zaneveld, J.R., Zhang, Y., Zhu, Q., Knight, R. and Caporaso, J.G. (2019) Reproducible, interactive, scalable and extensible microbiome data science using QIIME 2. Nature Biotechnology 37(8), 852-857.

Callahan, B.J., McMurdie, P.J., Rosen, M.J., Han, A.W., Johnson, A.J. and Holmes, S.P. (2016) DADA2: High-resolution sample inference from Illumina amplicon data. Nature Methods 13(7), 581-583.

Quast, C., Pruesse, E., Yilmaz, P., Gerken, J., Schweer, T., Yarza, P., Peplies, J. and Glockner, F.O. (2013) The SILVA ribosomal RNA gene database project: improved data processing and web-based tools. Nucleic Acids Research 41(Database issue), D590-596.

Ye, J., Coulouris, G., Zaretskaya, I., Cutcutache, I., Rozen, S. and Madden, T.L. (2012) Primer-BLAST: A tool to design target-specific primers for polymerase chain reaction. BMC Bioinformatics 13(1), 134.
