# Supplementary material for: Genome-centered metagenomics illuminates adaptations of core members to a partial Nitritation–Anammox bioreactor under periodic microaeration
Source: Front Microbiol. 2023 Jan 26;14:1046769. doi: 10.3389/fmicb.2023.1046769 (PMC9909701; doi:10.3389/fmicb.2023.1046769)
Supplement: Supplementary file 2 [file Presentation_1.PPTX]

## Slide 1
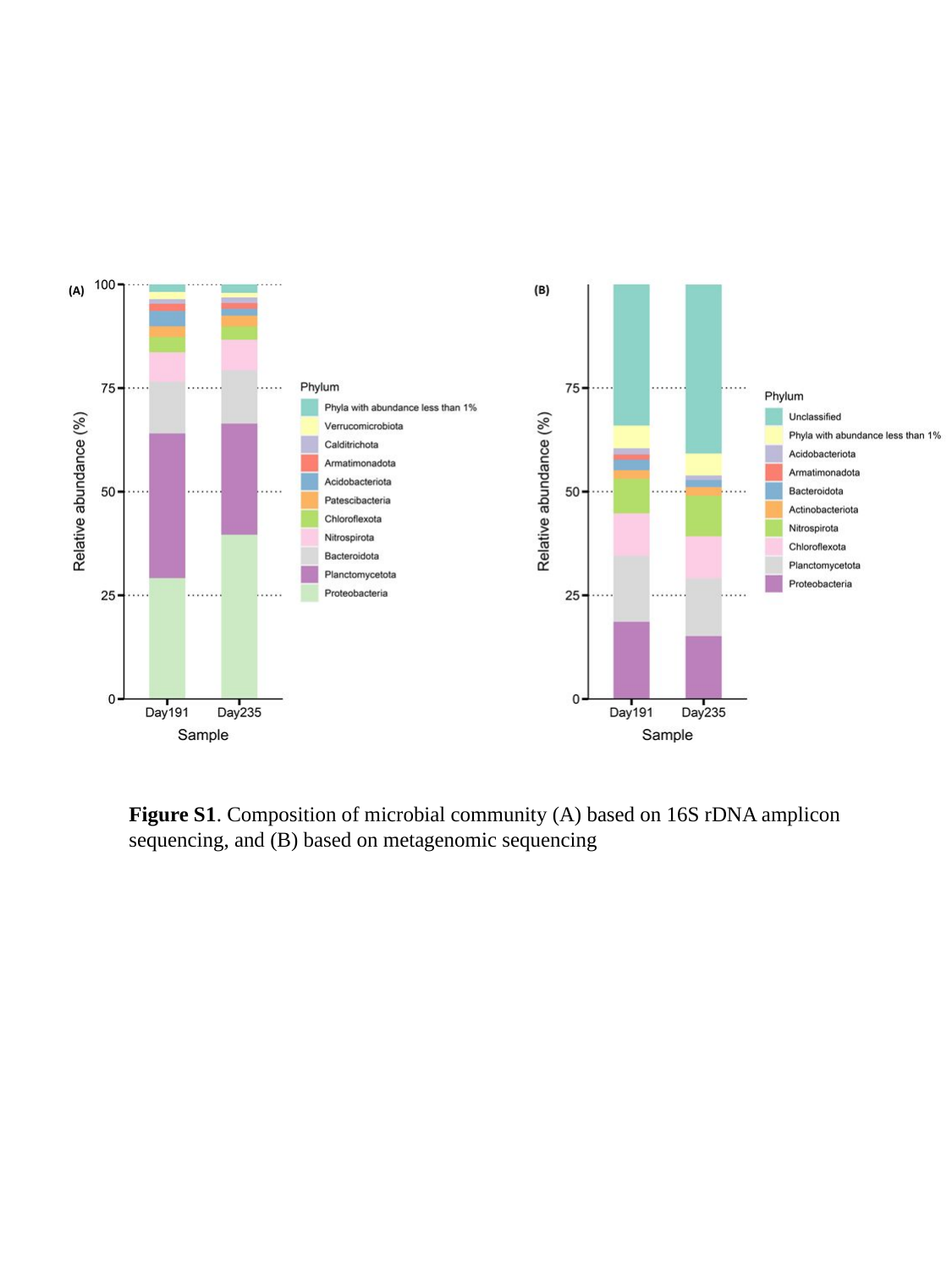

Figure S1. Composition of microbial community (A) based on 16S rDNA amplicon sequencing, and (B) based on metagenomic sequencing

## Slide 2
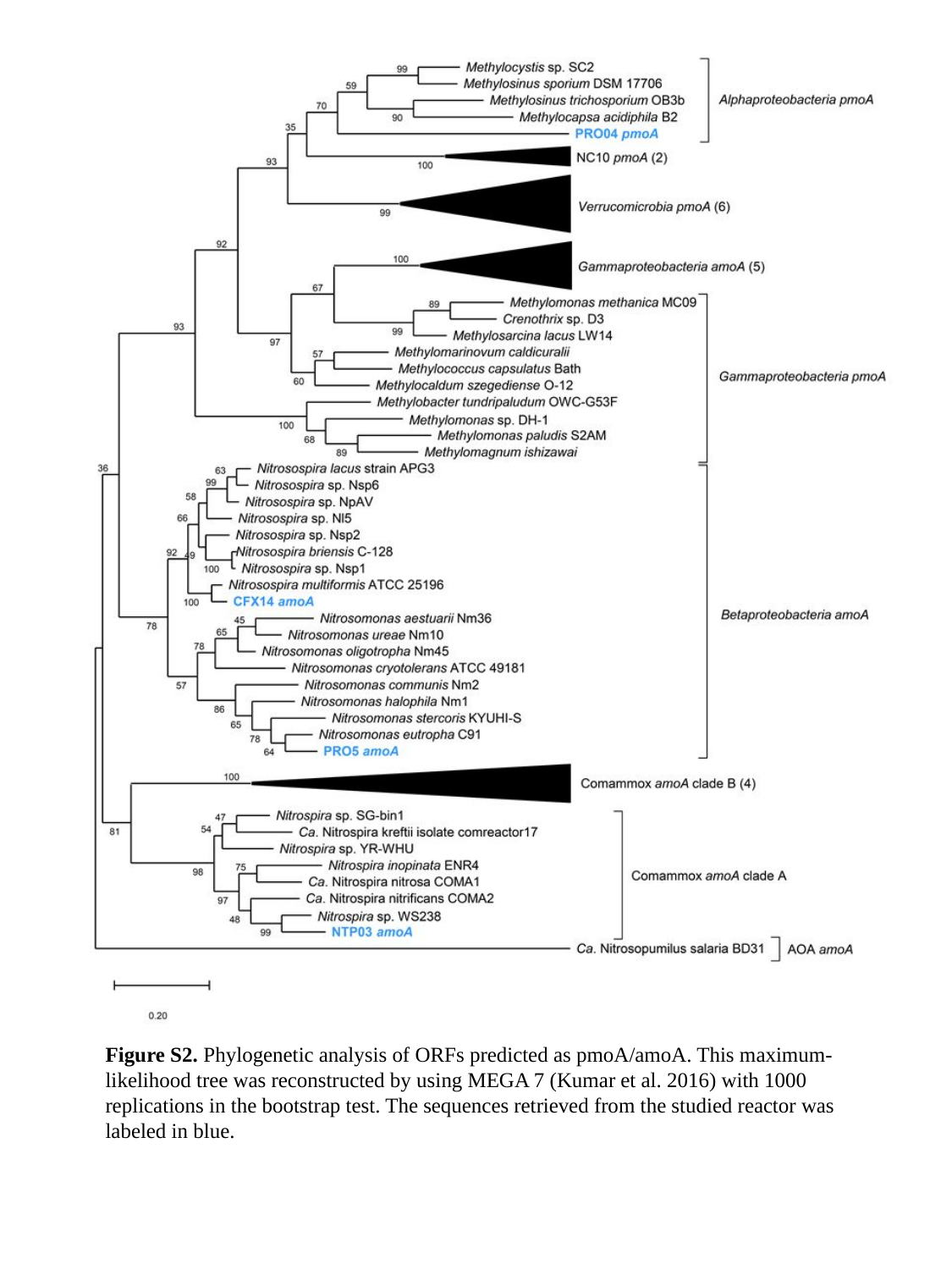

Figure S2. Phylogenetic analysis of ORFs predicted as pmoA/amoA. This maximum-likelihood tree was reconstructed by using MEGA 7 (Kumar et al. 2016) with 1000 replications in the bootstrap test. The sequences retrieved from the studied reactor was labeled in blue.

## Slide 3
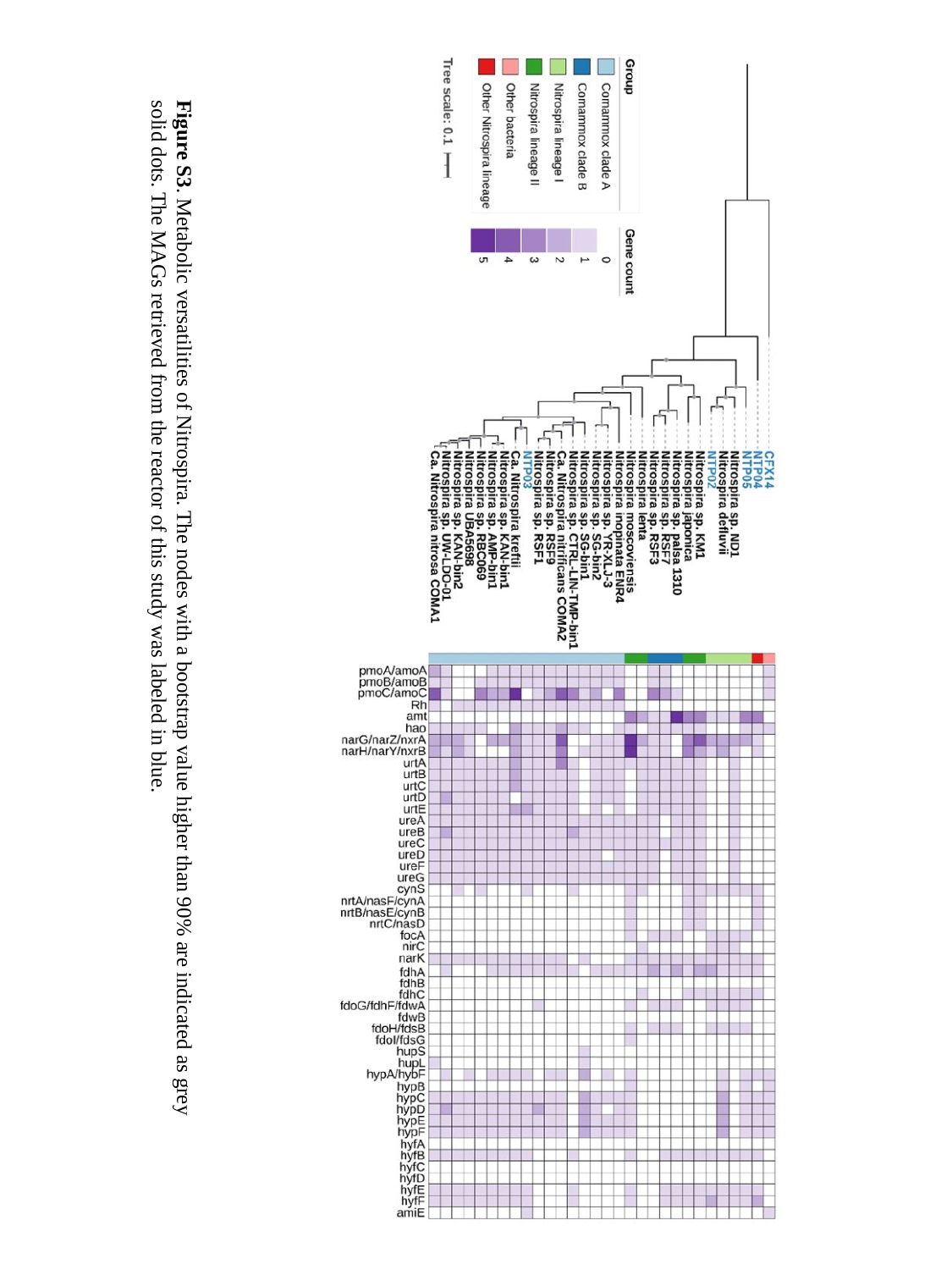

Figure S3. Metabolic versatilities of Nitrospira. The nodes with a bootstrap value higher than 90% are indicated as grey solid dots. The MAGs retrieved from the reactor of this study was labeled in blue.

## Slide 4
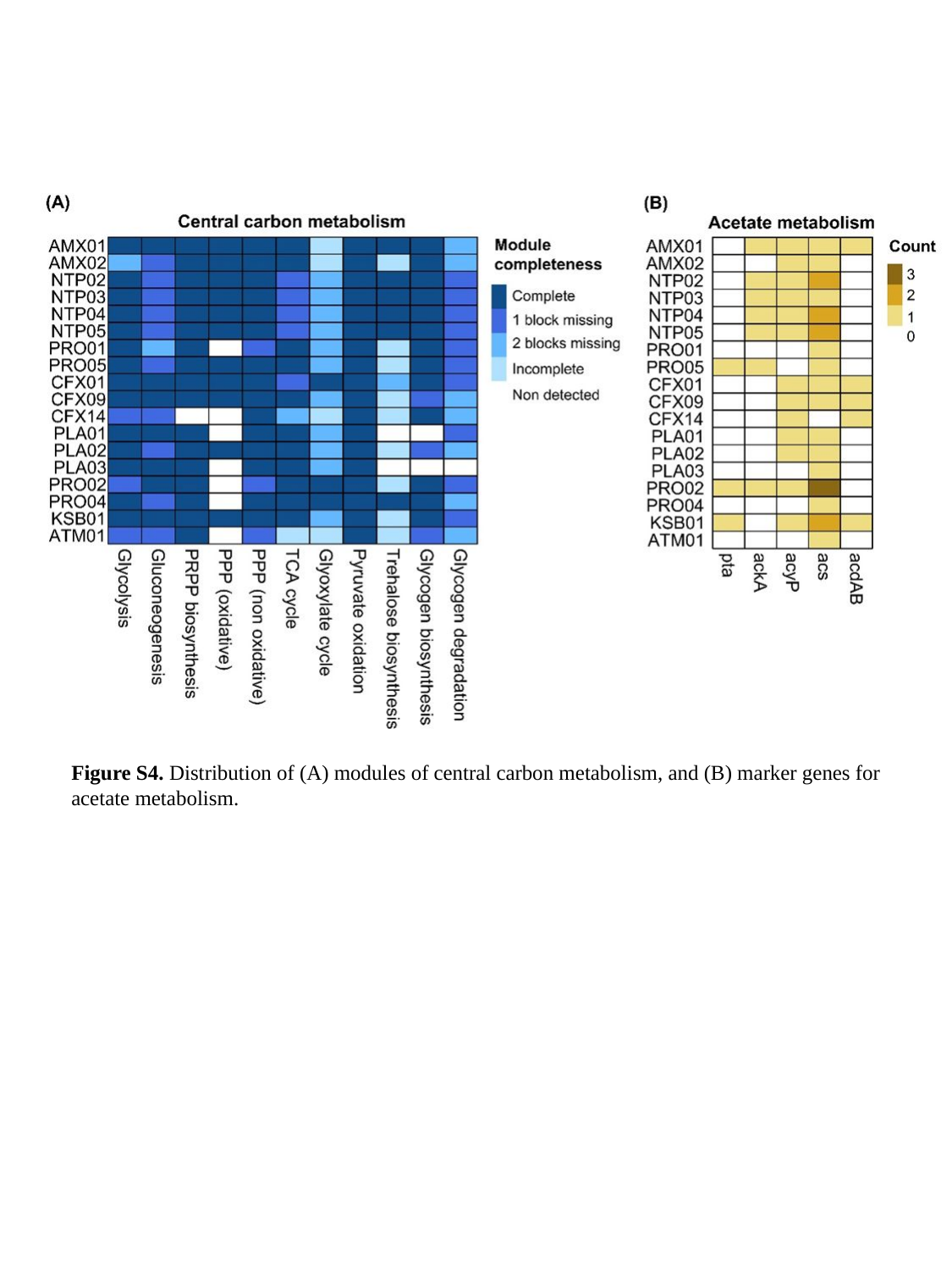

Figure S4. Distribution of (A) modules of central carbon metabolism, and (B) marker genes for acetate metabolism.

## Slide 5
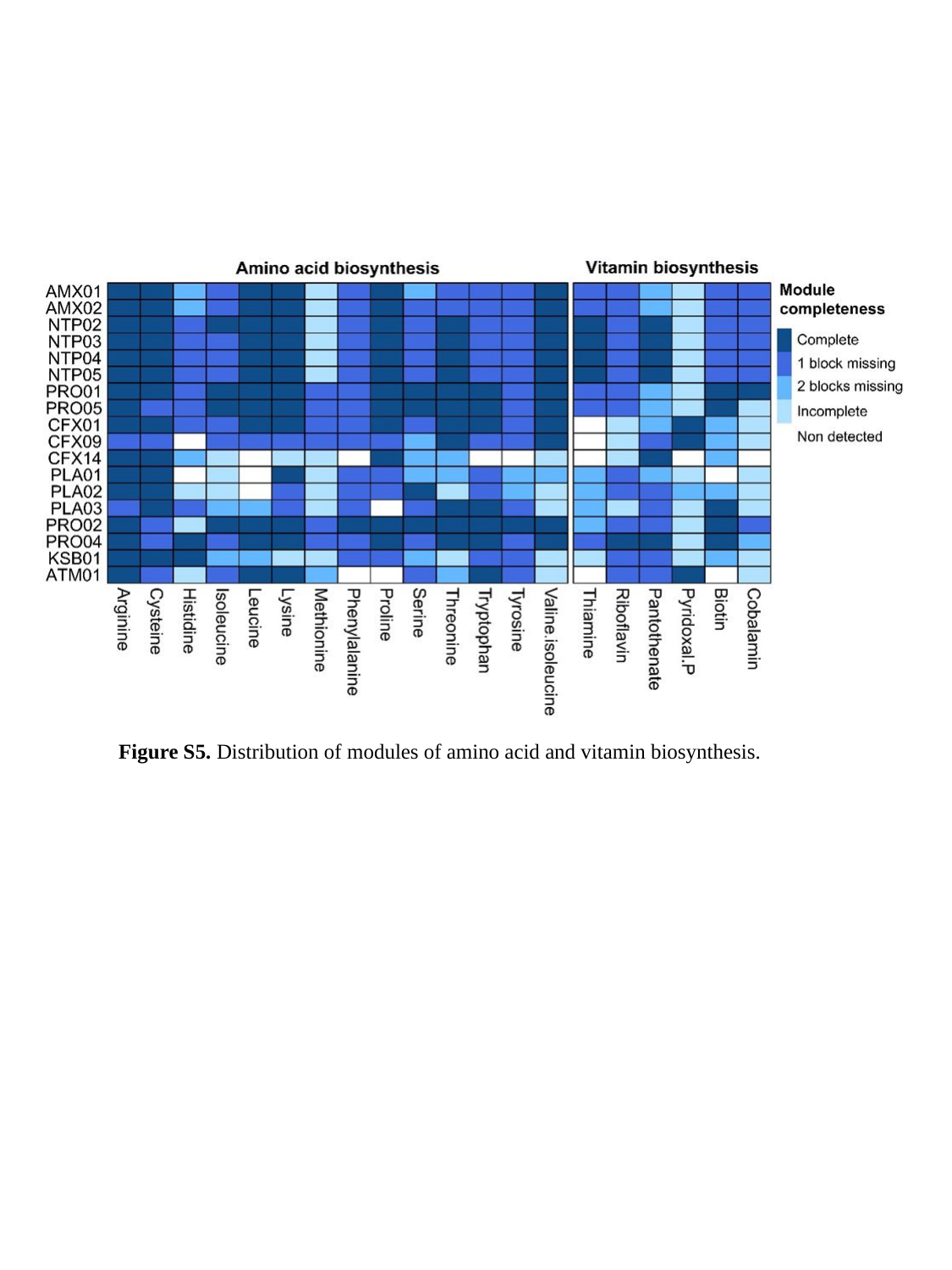

Figure S5. Distribution of modules of amino acid and vitamin biosynthesis.

## Slide 6
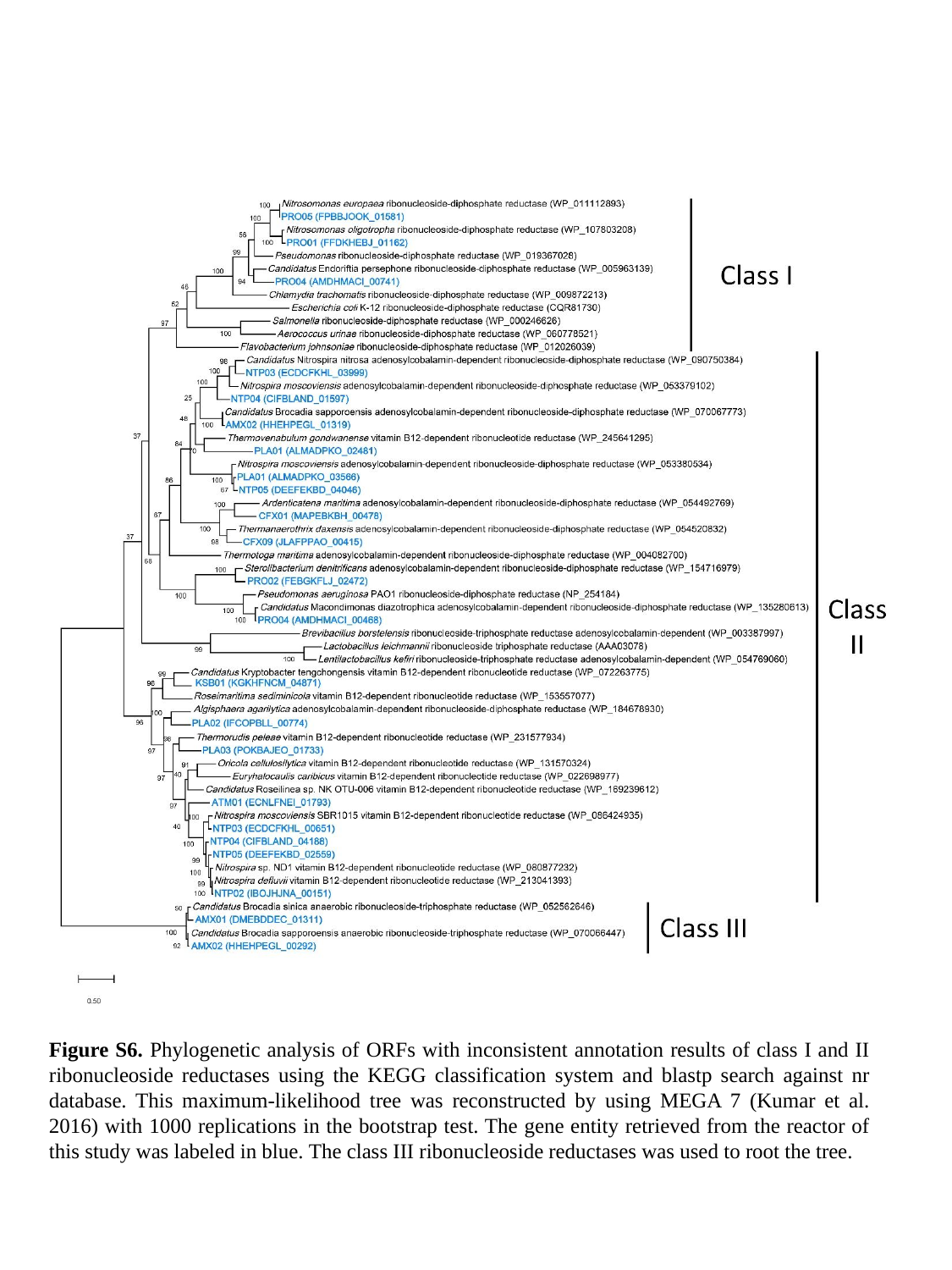

Figure S6. Phylogenetic analysis of ORFs with inconsistent annotation results of class I and II ribonucleoside reductases using the KEGG classification system and blastp search against nr database. This maximum-likelihood tree was reconstructed by using MEGA 7 (Kumar et al. 2016) with 1000 replications in the bootstrap test. The gene entity retrieved from the reactor of this study was labeled in blue. The class III ribonucleoside reductases was used to root the tree.

## Slide 7
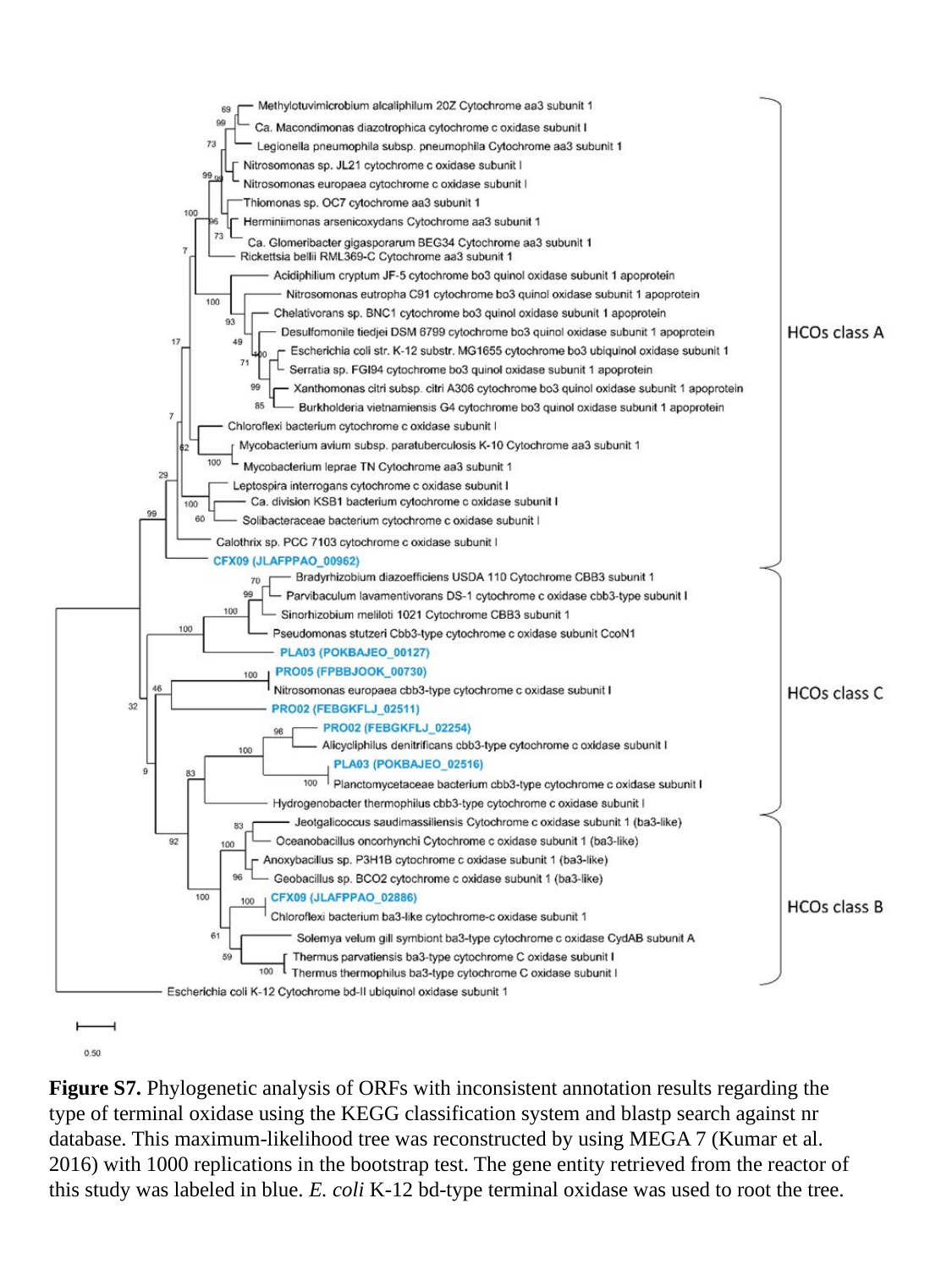

Figure S7. Phylogenetic analysis of ORFs with inconsistent annotation results regarding the type of terminal oxidase using the KEGG classification system and blastp search against nr database. This maximum-likelihood tree was reconstructed by using MEGA 7 (Kumar et al. 2016) with 1000 replications in the bootstrap test. The gene entity retrieved from the reactor of this study was labeled in blue. E. coli K-12 bd-type terminal oxidase was used to root the tree.
